# Supplementary material for: DNMT1-mediated regulation of somatostatin-positive interneuron migration impacts cortical architecture and function
Source: Nat Commun. 2025 Jul 24;16:6834. doi: 10.1038/s41467-025-62114-0 (PMC12290092; doi:10.1038/s41467-025-62114-0)
Supplement: Supplementary file 1 — Supplementary Information File [file 41467_2025_62114_MOESM1_ESM.pdf]

Supplementary Information

DNMT1-Mediated Regulation of Somatostatin-positive Interneuron Migration Impacts Cortical Architecture and Function

Julia Reichard[1,2*], Philip Wolff[1,2*], Song Xie[3,4], Ke Zuo[3,5,6], Camila L. Fullio[7,8], Jian Du[1], Severin Graff[2,9], Jenice Linde[1,2], Can Bora Yildiz[1,2], Georg Pitschelatow[1,2], Gerion Nabbefeld[2,10], Lilli Dorp[1], Johanna Vollmer[1], Linda Biemans[1], Shirley Kempf[1], Minali Singh[11], K. Naga Mohan[11], Chao-Chung Kuo[12], Tanja Vogel[7], Paolo Carloni[3,4], Simon Musall[2,9] and Geraldine Zimmer-Bensch[1, 2*]

[1] RWTH Aachen University, Division of Neuroepigenetics, Institute of Zoology (Biology 2), Worringerweg 3, 52074 Aachen, Germany.

[2] Research Training Group 2416 MultiSenses – MultiScales, RWTH Aachen University, 52074 Aachen, Germany

[3] Institute of Neuroscience and Medicine (INM-9) Computational Biomedicine, Forschungszentrum Jülich GmbH, 52428 Jülich, Germany

[4] RWTH Aachen University, Department of Physics, 52074 Aachen, Germany

[5] College of Pharmacy (International Academy of Targeted Therapeutics and Innovation), Chongqing University of Arts and Sciences, 402160 Chongqing, PR China

[6] Department of Physics, University of Cagliari, I-09042 Cagliari, Italy

[7] Institute for Anatomy and Cell Biology, Department of Molecular Embryology, Faculty of Medicine, Albert-Ludwigs-University Freiburg, 79104 Freiburg, Germany

[8] Faculty of Biology, Albert-Ludwigs-University Freiburg, 79104 Freiburg, Germany

[9] Institute of Biological Information Processing (IBI-3) Bioelectronics, Forschungszentrum Jülich, Germany

[10] RWTH Aachen University, Division of Neurophysiology, Institute of Zoology (Biology 2), Worringerweg 3, 52074 Aachen, Germany.

[11] Molecular Biology and Genetics Laboratory, Department of Biological Sciences, BITS Pilani, Hyderabad Campus, Hyderabad, India

[12] Genomics Facility, Interdisciplinary Center for Clinical Research (IZKF), RWTH Aachen University, 52074 Aachen, Germany

1. Supplementary Figures

Supplementary Figure S1

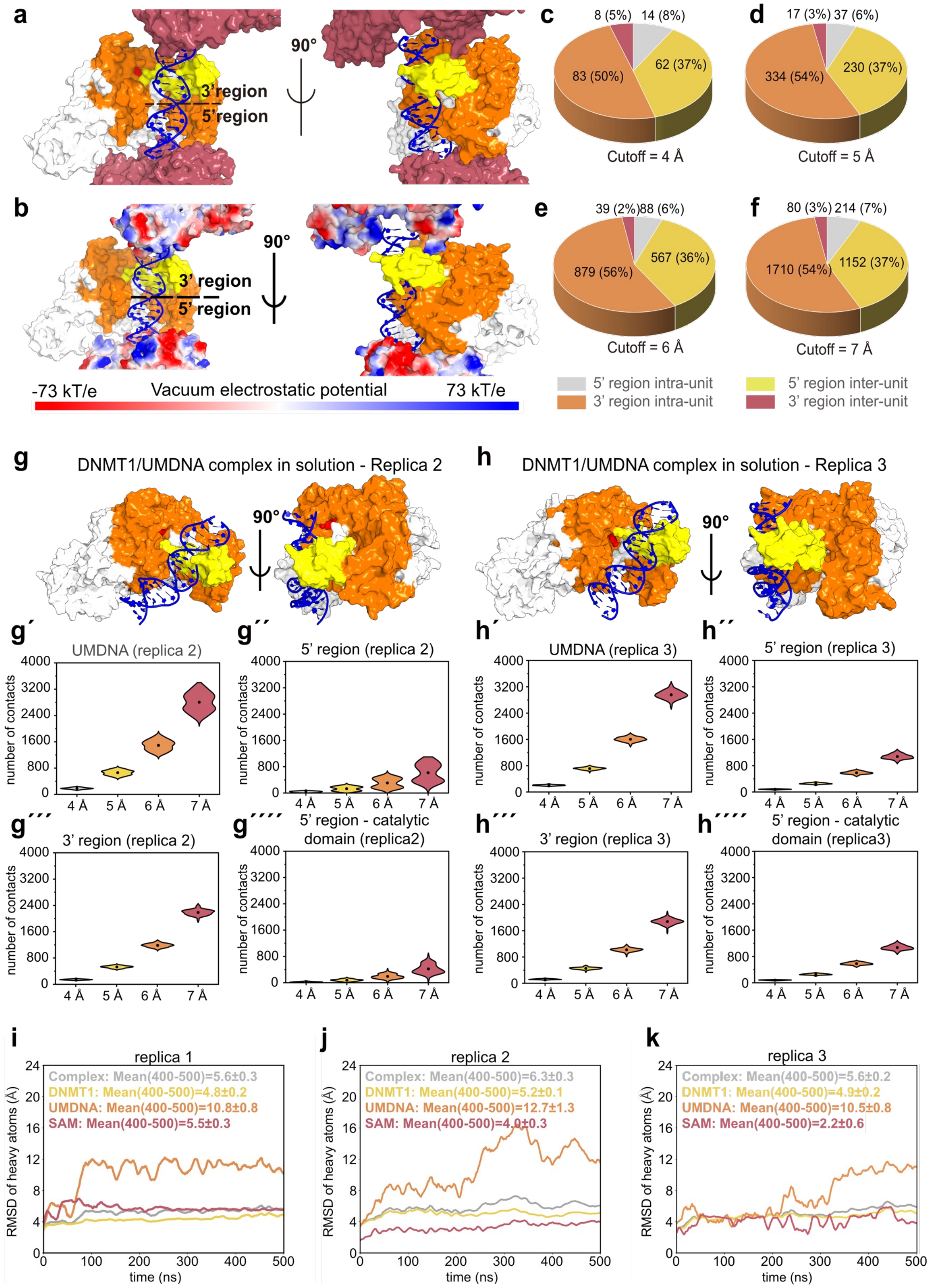

Supplementary Figure S2

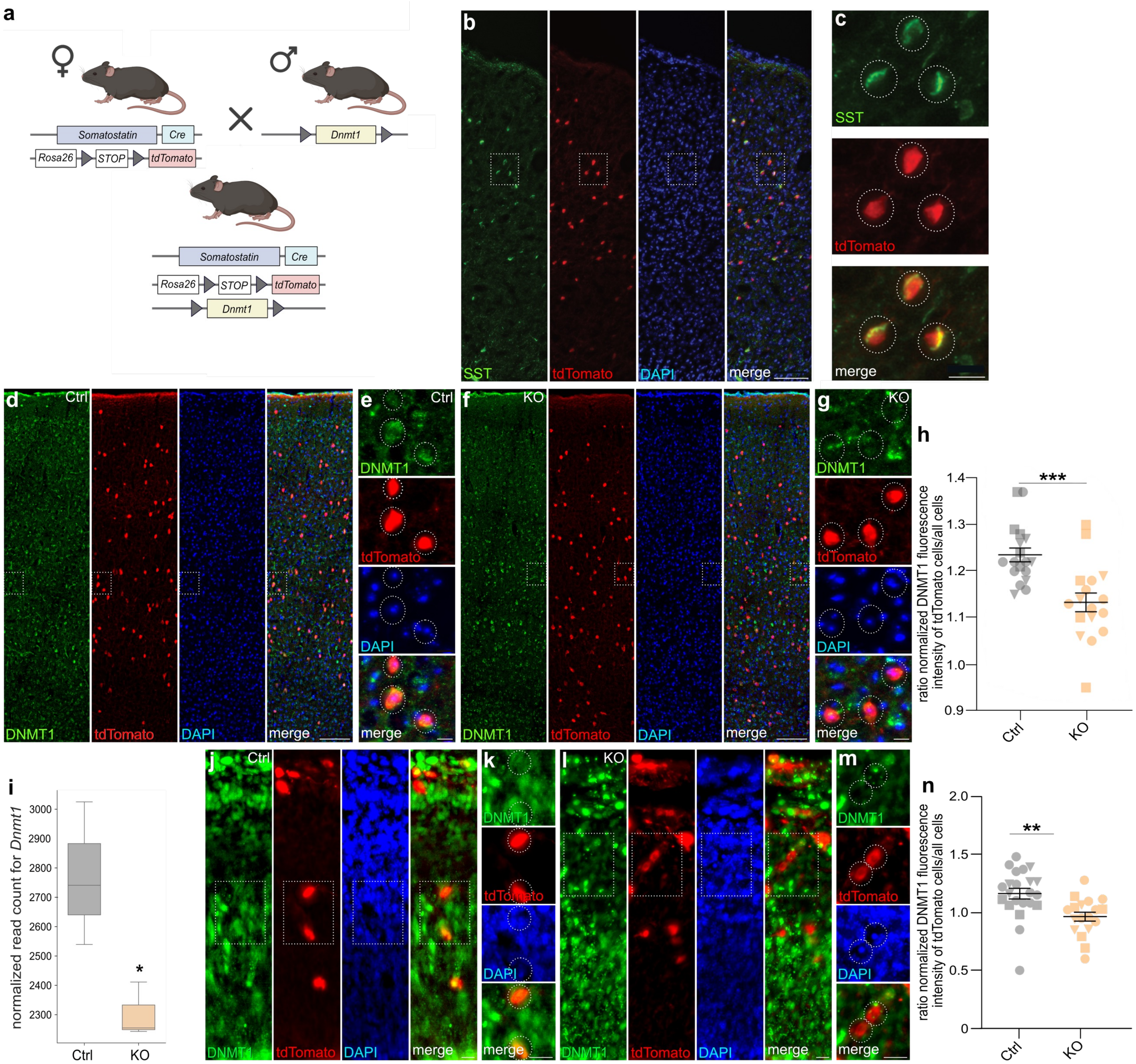

Supplementary Figure S3

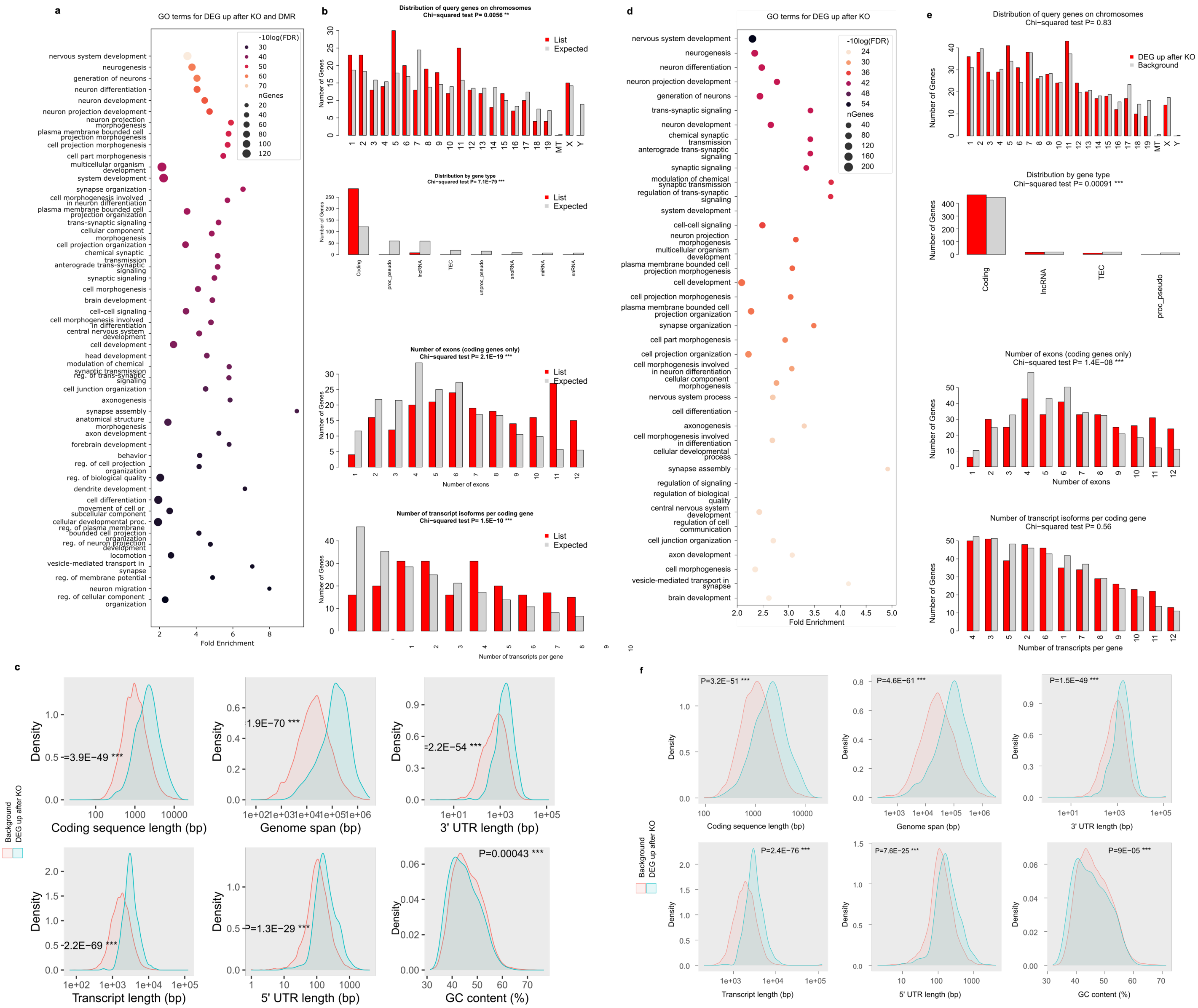

Supplementary Figure S4

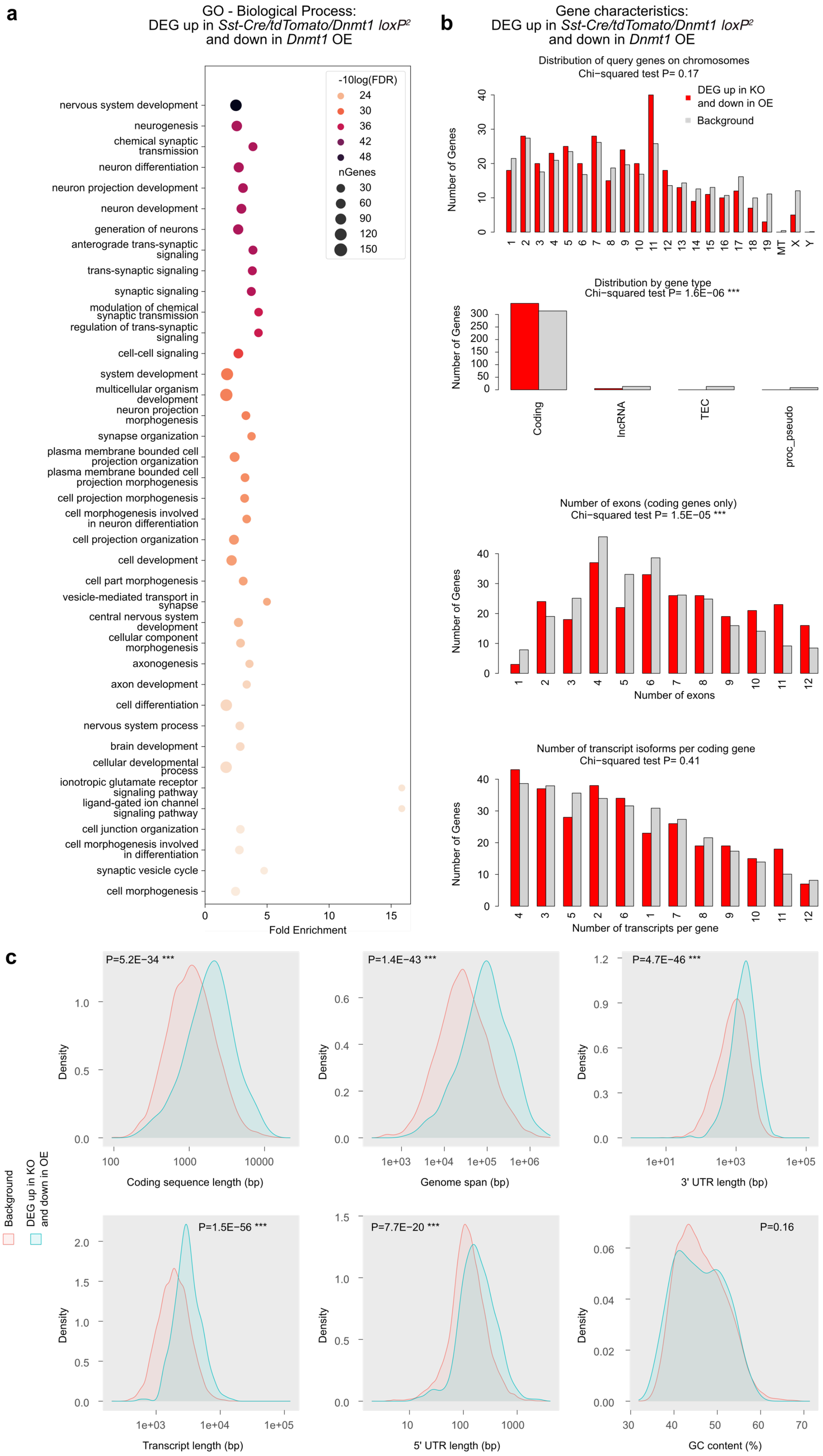

Supplementary Figure S5

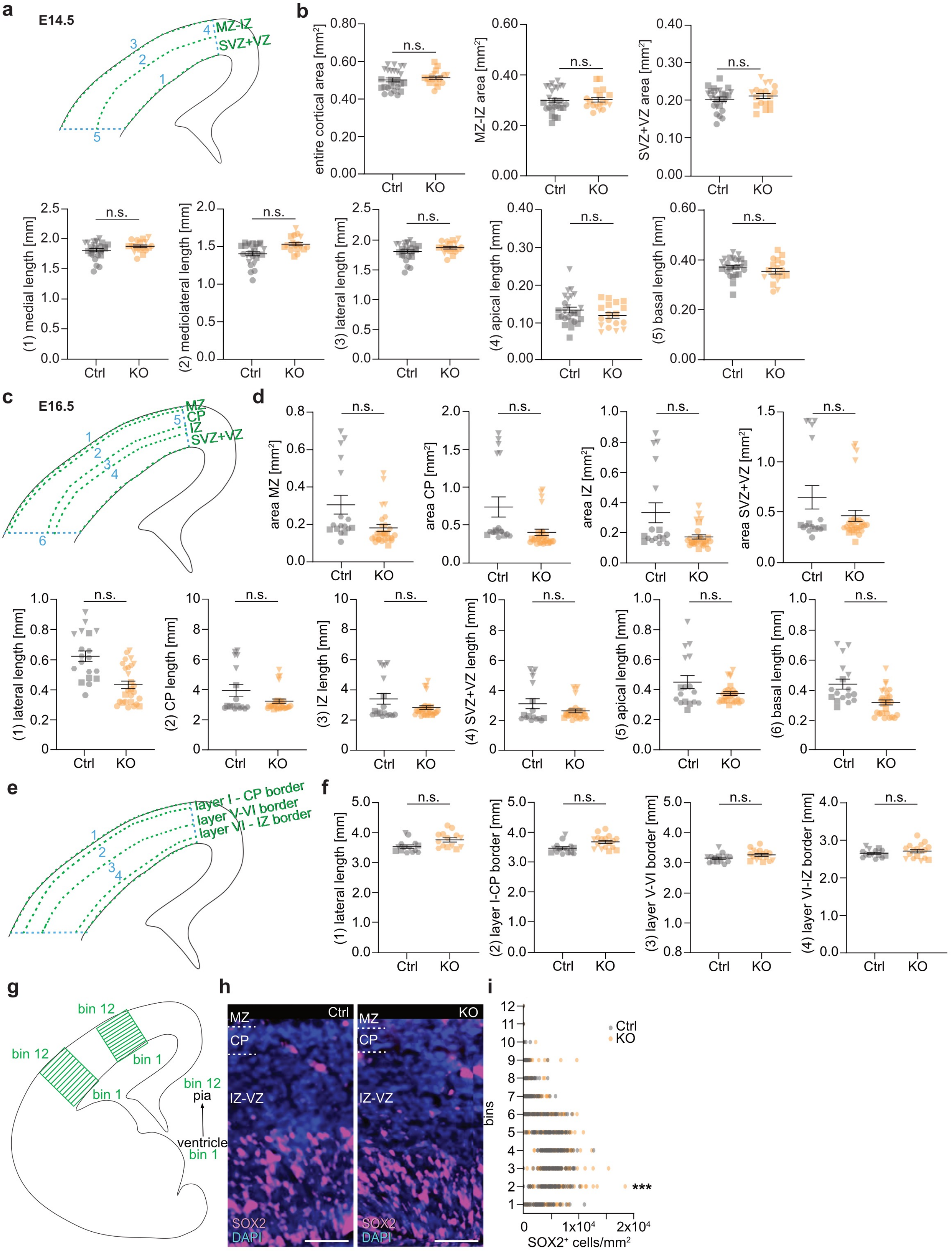

Supplementary Figure S6

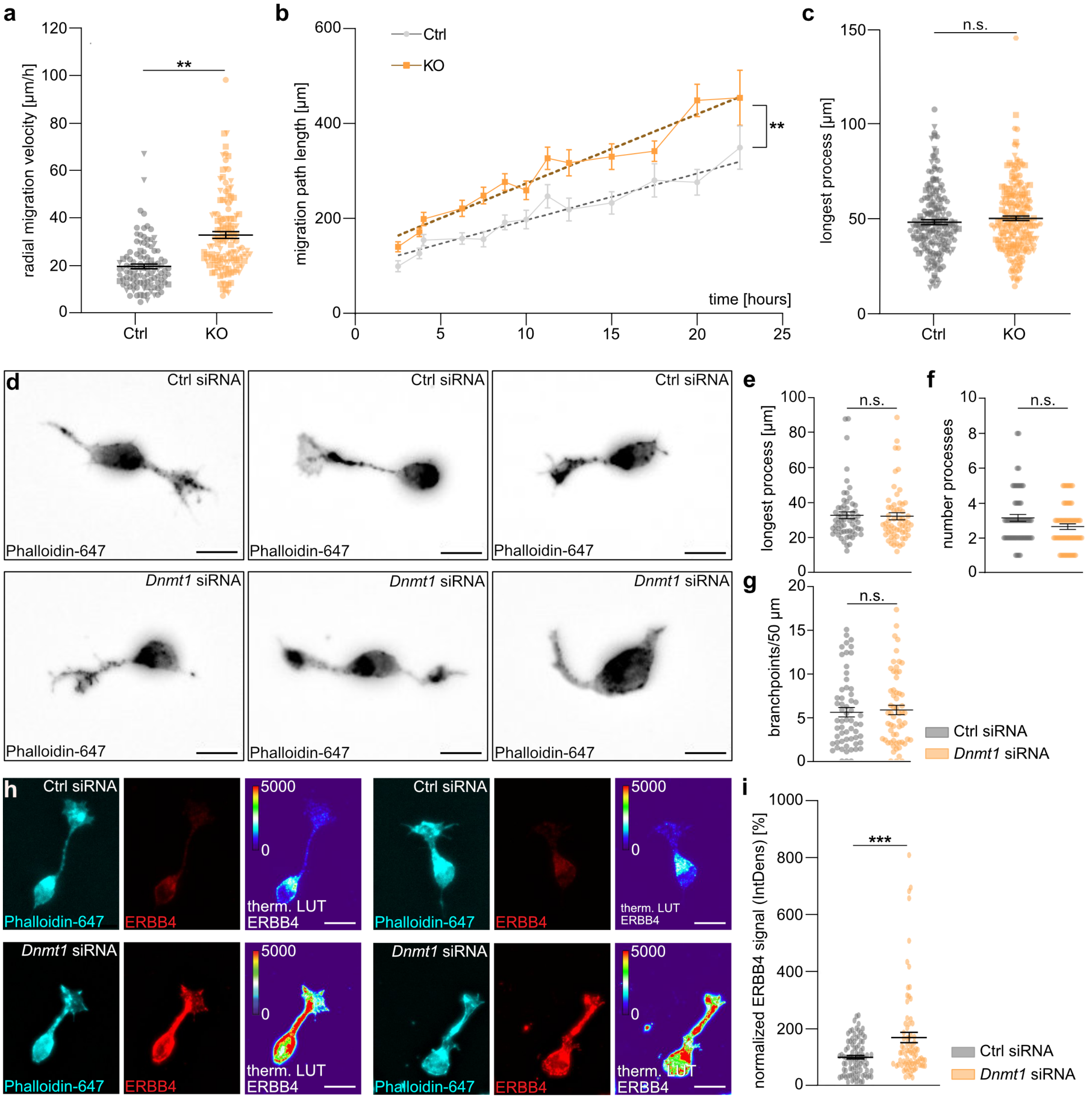

Supplementary Figure S7

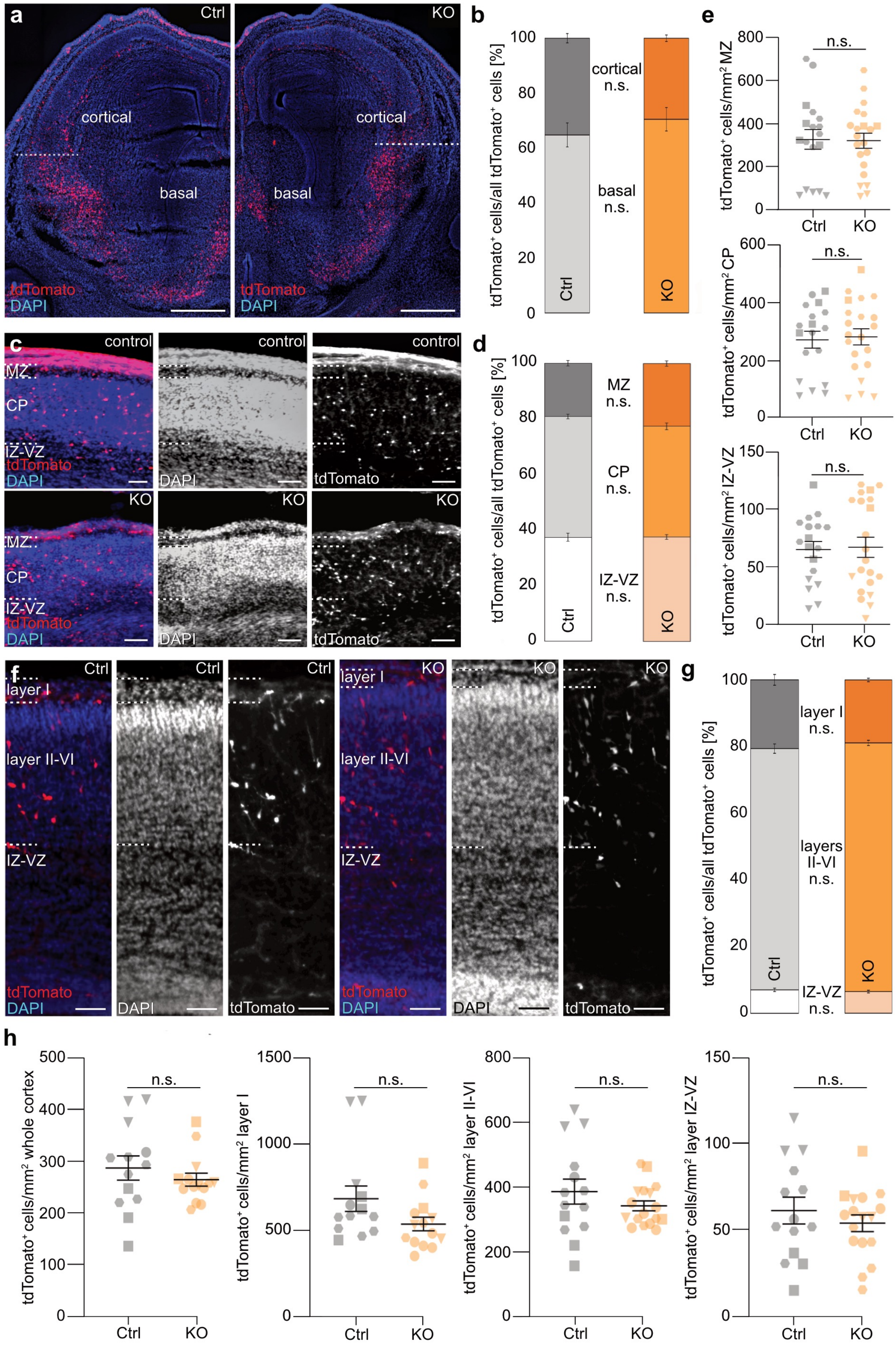

Supplementary Figure S8

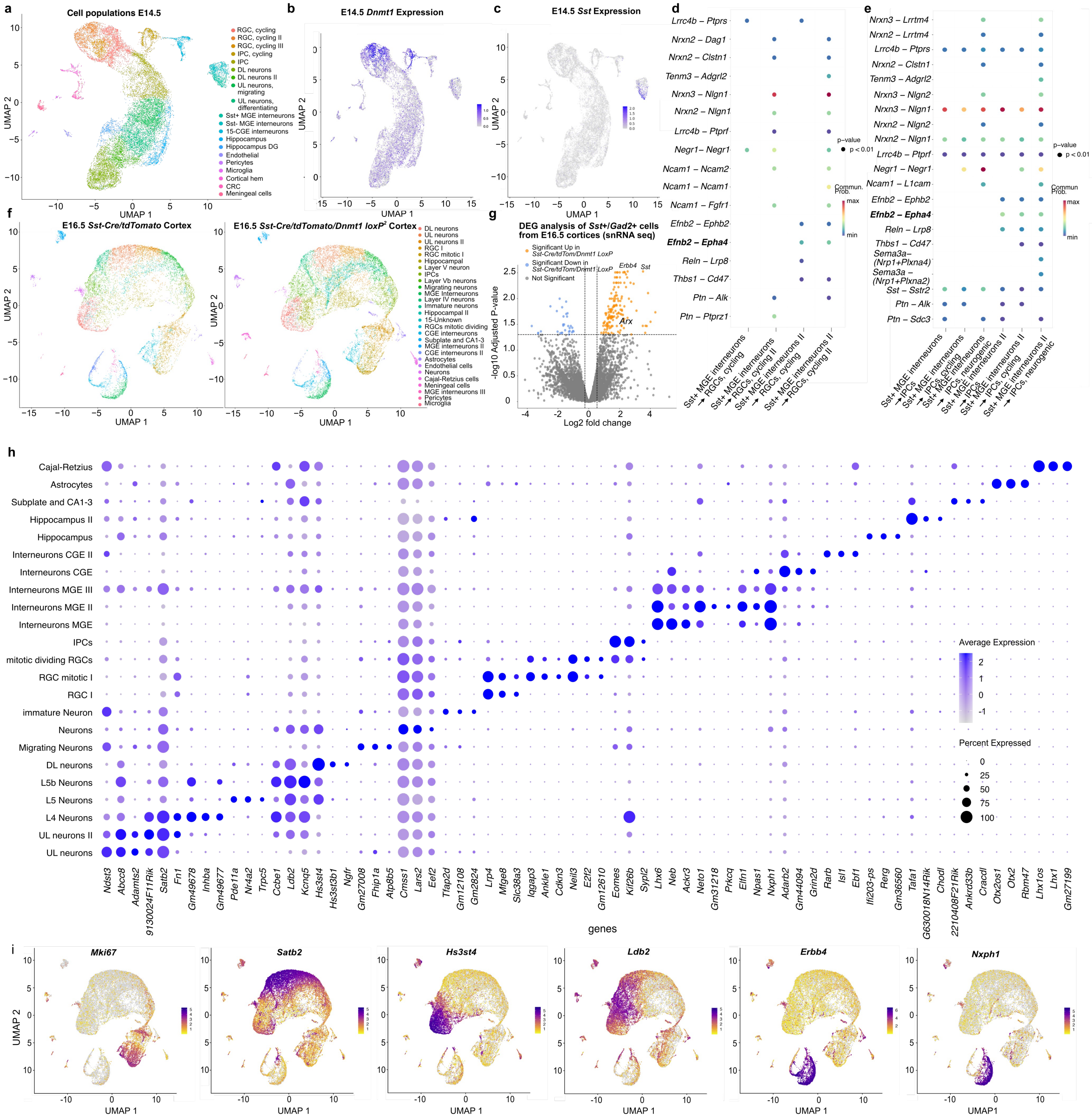

Supplementary Figure S9

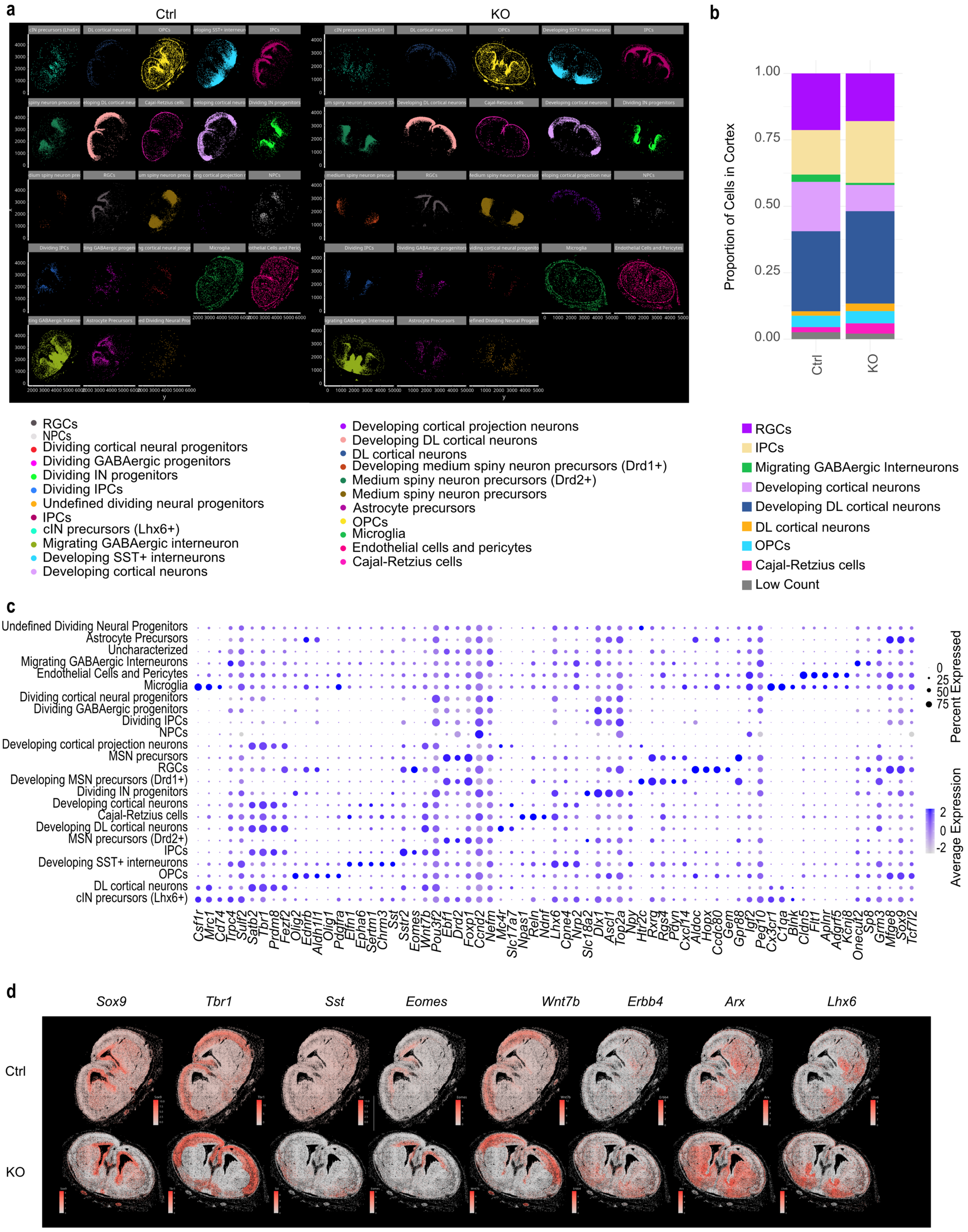

Supplementary Figure S10

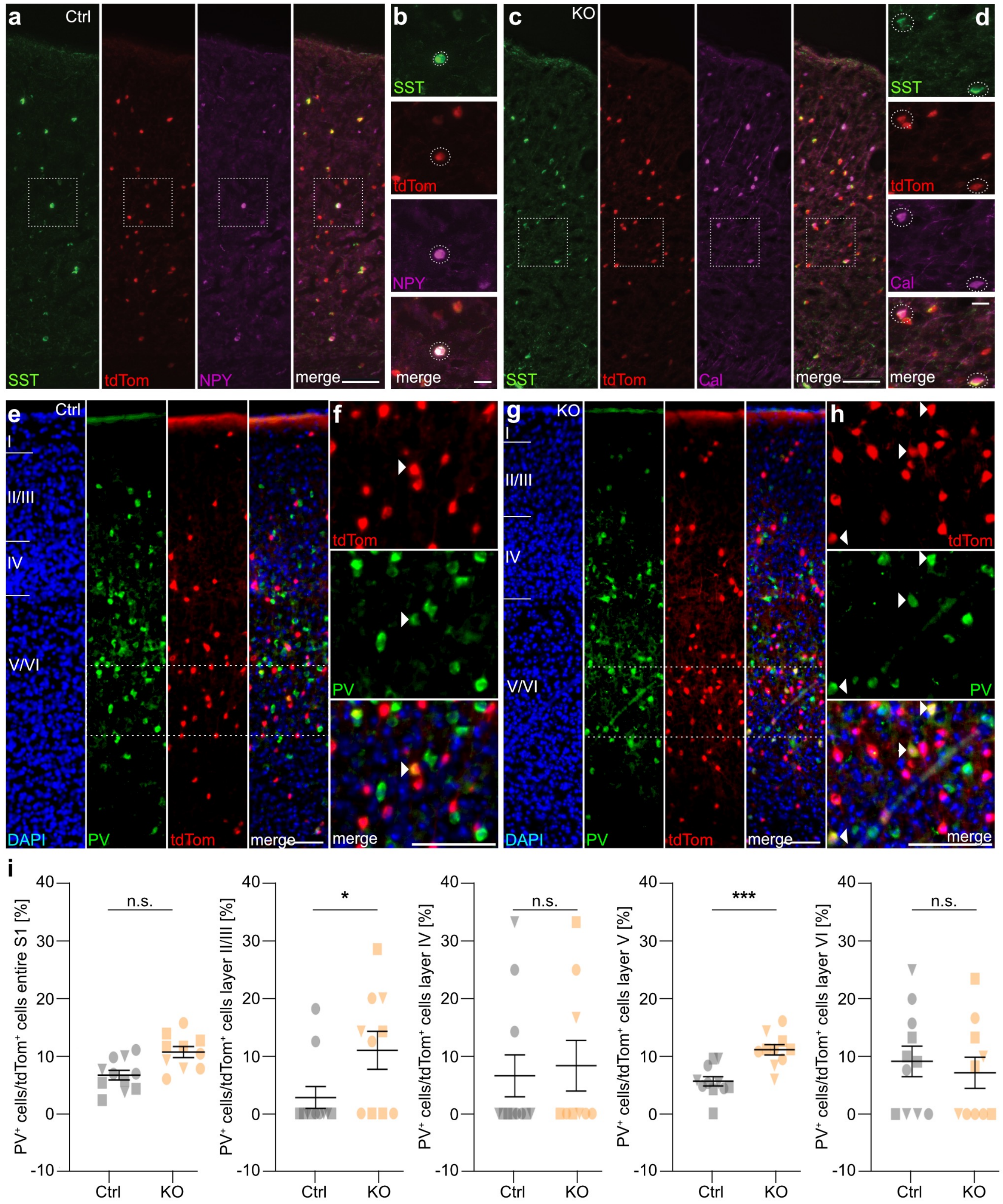

Supplementary Figure S11

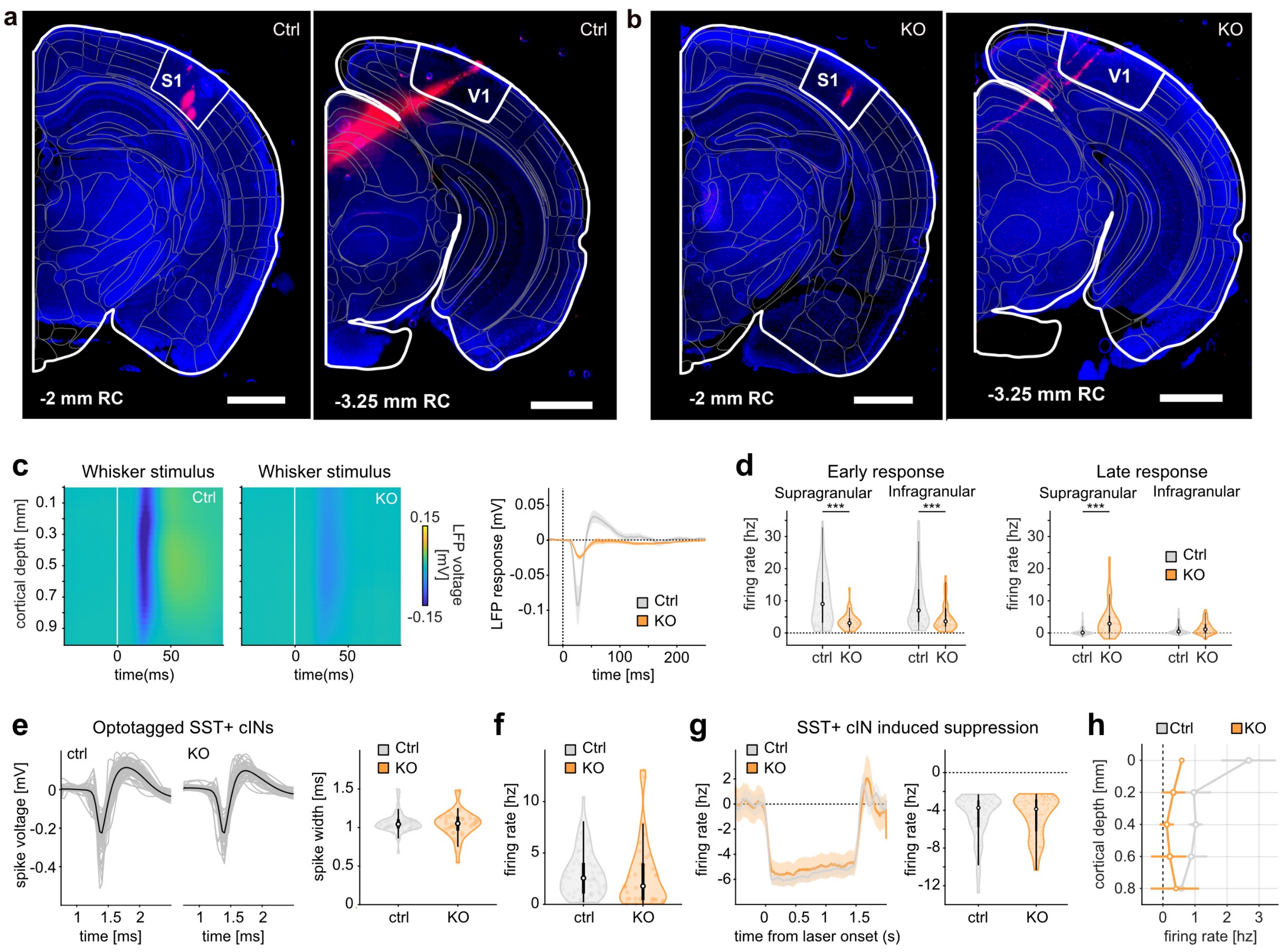

Supplementary Figure S12

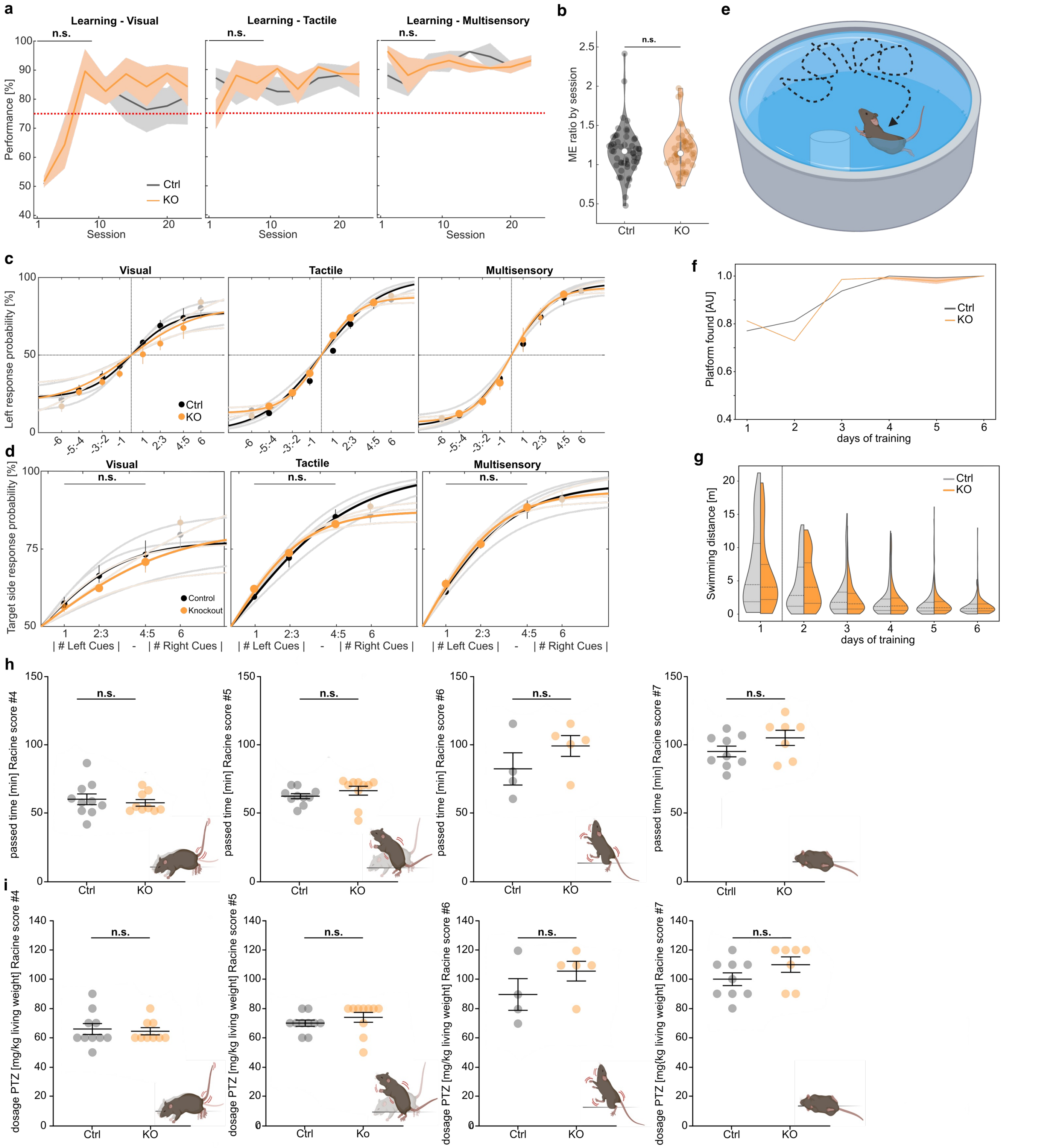

2. Supplementary Figure Legends

Supplementary Figure S1:

Molecular dynamics simulations of DNMT1 binding to unmethylated DNA (UMDNA).
(a-f) Inter-unit DNMT1 proteins interacting with the DNMT1/UMDNA/SAH complex unit in the X-ray structure (PDB ID: 3PTA). **(a)** Intra-unit DNMT1 regions shown including the CXXC domain (yellow), which interacts most with UMDNA (blue), autoinhibitory linker BAH1/BAH2 (white surface), catalytic domain (orange), and SAH (red spheres). The neighboring inter-unit DNMT1 proteins are illustrated in rose-pink. **(b)** Electrostatic surface representation of the complex observed in the X-ray structure. Surfaces are colored as in **(a)**. The additional red and blue surfaces represent negative and positive potential regions, respectively. The inter-unit DNMT1 regions that the UMDNA interacts with are mostly positively charged. **(c-f)** Number of contacts of the 5' and 3' regions with their intra-unit and inter-unit DNMT1 with different distance cutoffs in the X-ray structure. **(g, h)** Simulations of DNMT1/UMDNA interactions in aqueous solution. The complexes shown are at the end of replica 2 and replica 3 with the same coloring scheme as **(a)**. Number of contacts between DNMT1 with UMDNA **(g´, h´)**, 5' regions **(g´´, h´´)**, and 3' regions **(g´´´, h´´´)** as well as between 5' region and the catalytic domain of DNMT1 **(g´´´´, h´´´´)** during last 100 ns simulations.
(i-k) The heavy-atom root-mean-square deviation (RMSD) values for each component were monitored across three independent MD simulations with (**i**) depicting replica 1, **(j)** depicting replica 2, and **(k)** depicting replica 3. UMDNA exhibited significant conformational changes in all replicas, with RMSD values exceeding 10 Å. Raw data are available via the hyperlinks listed in the Data Availability Statement.

Supplementary Figure S2:

Validation of SST expression and reduced DNMT1 levels in tdTomato⁺ cells. **(a)** Mating strategy to obtain *Sst-Cre/tdTomato* (Ctrl) and *Sst-Cre/tdTomato/Dnmt1 loxP*^{2} (KO) mice (see methods and supplementary methods for detailed information). **(b)** Microphotographs depicting SST expression in tdTomato-cells in sagittal brain sections (4-month-old male Ctrl mouse, Bregma 2.76). **(c)** Magnified images of the areas outlined in (**b**). Scale bars: 100 µm in (**b**) and 20 µm (**c**). **(d-g)** Quantification of DNMT1 expression by immunohistochemistry in Ctrl **(d, e)** and KO **(f, g)** male mice (6month-old) in 30 µm, fixated coronal cryosections (Bregma 1.18, 1.10, 0.14, -0.22, -2.92. -3.08, and -3.28). Scale bars **(d, f)** 100 µm and 10 µm **(e, g)**. **(h)** Quantified ratio of the normalized mean grey value of the DNMT1 immunofluorescence signal in *tdTomato*$^{+}$ cells to the normalized mean grey value of DNMT1 immunofluorescence of all cortical nuclei (identified by DAPI, blue). Statistical testing via nested two-way ANOVA ($p < 0.001$ ***, $N = 3$ for Ctrl ($n = 18$ slices) and KO ($n = 17$ slices)). **(i)** *Dnmt1* transcript counts determined by RNA sequencing of FAC-sorted Ctrl and KO cells from the E14.5 cortex. 3 libraries were analyzed for both genotypes from pooled material of $n = 7$ Ctrl embryos, and $n = 8$ KO embryos; $log_2FC = -0.26482506$, p_{adj}: 0.0121078). **(j-n)** Quantification of DNMT1 expression by immunohistochemistry in *Dnmt1* Ctrl **(j, k)** and KO **(l, m)** E14.5 embryos in 50 µm, fixated coronal cryosections. Scale bars 50 µm in **(j, l)** and 25 µm **(k, m)**. **(n)** Quantified ratio of the normalized mean grey value of the DNMT1 immunofluorescence signal in tdTomato^{+} cells to the normalized mean grey value of DNMT1

immunofluorescence of all cortical nuclei (identified by DAPI, blue). Statistical testing via nested two-way ANOVA ($p < 0.01$ **, $N = 3$ for Ctrl ($n = 22$ slices) and KO ($n = 18$ slices)). Data points originating from the same mouse are plotted using the same symbol. Error bars in (h) and (n): +/- SEM of the mean. Boxplots in (i) represent the interquartile range (IQR), with the median. Moreover, the 25th percentile (Q1) and the 75th percentile (Q3) are shown by horizontal lines and whiskers extend to the data's minimum and maximum values. Exact p-values are provided by Supplementary Data 10. Raw data are available via the hyperlinks listed in the Data Availability Statement. FC = foldchange, KO = knockout, Ctrl. = control.

Supplementary Figure S3:
RNA Sequencing and methyl-sequencing analysis of FAC-sorted *Sst-Cre/tdTomato/Dnmt1 loxP²* neurons compared to *Sst-Cre/tdTomato* control cells from the E14.5 basal telencephalon

(a-c) Gene ontology (GO) analysis and characteristics of genes that were upregulated in expression and differentially methylated in FAC-sorted *Sst-Cre/tdTomato/Dnmt1 loxP²* neurons compared to *Sst-Cre/tdTomato* control cells from the E14.5 basal telencephalon. Background is defined as all detected transcripts in both genotypes; Chi-squared and Student's *t*-tests were run to analyze if our gene set has special characteristics when compared with all the other genes (ShinyGO 0.82; http://bioinformatics.sdstate.edu/go/). **(a)** Gene ontology (GO) analysis – Biological Process, **(b)** bar plots (tested by Chi-squared test), and **(c)** density plots (Student's *t*-test). **(d-f)** GO analysis and characteristics of genes that were upregulated in expression in FAC-sorted *Sst-Cre/tdTomato/Dnmt1 loxP²* neurons compared to *Sst-Cre/tdTomato* cells prepared from the E14.5 basal telencephalon. Background is defined as all detected transcripts in both genotypes; Chi-squared and Student's *t*-tests were run to analyze if our gene set has special characteristics when compared with all the other genes (ShinyGO 0.80; http://bioinformatics.sdstate.edu/go/). **(d)** GO analysis - Biological Process, **(e)** bar plots (tested by Chi-squared test) and **(f)** density plots (Student's *t*-test) that depict the characteristics of this gene set compared with the entire set of detected transcripts. Raw data are available via the hyperlinks listed in the Data Availability Statement and Supplementary Tables 1-7.

Supplementary Figure S4:
Analysis of genes overlapping between upregulated genes in *Sst-Cre/tdTomato/Dnmt1-loxP²* cells and downregulated after *Dnmt1* overexpression in neurons obtained from ESCs

(a-c) Gene ontology (GO) analysis and characteristics of genes that are upregulated in *Sst* expressing interneurons of *Sst-Cre/tdTomato/Dnmt1-loxP²* mice and overlap with genes that are downregulated after *Dnmt1* overexpression (OE) in neurons obtained from murine embryonic stem cells (ESCs)[1]. **(a)** GO analysis (ShinyGO 0.80; http://bioinformatics.sdstate.edu/go/) for the overlap. **(b)** Further characteristics are shown in bar plots (tested by Chi-squared test) and density plots (Student's *t*-test) **(c)**, depicting the characteristics of this gene set compared with the entire set of detected transcripts. Exact *p*-values and raw data are Raw data are available via the hyperlinks listed in the Data Availability Statement and Supplementary Tables 1-7.

Supplementary Figure S5:

The cortical size and dimensions of its different regions do not differ between *Sst-Cre/tdTomato/Dnmt1-loxP*^{2} (KO) and *Sst-Cre/tdTomato* (Ctrl) brains at E14.5, E16.5, and E18.5. (a) Scheme of cortical regions and their extensions in a hemisphere of 50 µm-thick coronal brain sections from E14.5 embryos. Quantifications of different parameters are depicted in **(b)**. Quantification was conducted by using a nested two-way ANOVA with $N = 3$ embryos for both genotypes, $n = 13$ slices for Ctrl, and $n = 9$ slices for KO samples. **(c)** and **(e)** illustration of cortical regions and their extensions in a single hemisphere of 50 µm-thick coronal brain sections from E16.5 and E18.5 embryos, respectively. The cortical size and dimensions for E16.5 and E18.5 KO embryos compared to age-matched control individuals are quantified in **(d)** for E16.5 and in **(f)** for E18.5 (nested two-way ANOVA with $N = 4$ embryos for both genotypes, $n = 12$ slices for Ctrl, and $n = 15$ slices for KO for E16.5 **(d),** and $N = 4$ embryos for both genotypes, $n = 12$ slices for Ctrl, and $n = 9$ slices for KO embryos at 18.5 **(f)**)**. (g-i)** SOX2 Immunostaining in E14 coronal sections of KO embryos and age-matched Ctrl embryos. **(g)** Scheme of a coronally sectioned embryonic brain hemisphere illustrating the lateral and dorsal cortical regions that were analyzed regarding the density and distribution of SOX2^{+} cells. **(h)** Immunhistochemical staining of SOX2 in coronal sections (50 µm) of E14.5 Ctrl and KO embryos (DAPI: blue and SOX2: magenta). Scale bars: 50 µm. **(i)** Quantitative analysis of SOX2^{+} cells per bin (nested two-way ANOVA. $p < 0.05$ *, $p < 0.01$ **, $p < 0.001$ ***; $n = 17$ Ctrl and $n = 14$ KO sections from $N = 5$ embryos for both genotypes). MZ: marginal zone, CP: cortical plate, IZ: intermediate zone, SVZ+VZ: subventricular zone, n.s.: not significant, KO = knockout, Ctrl = control. Data points with the same symbol are from the same embryo. Error bars: +/- SEM of the mean (detailed information is provided by Supplementary Data 10).

Supplementary Figure S6:

DNMT1 regulates ERBB4 expression and proper migration of MGE-derived cells but does not affect morphology. (a-c) Live-cell imaging analysis of *Sst-Cre/tdTomato* (Ctrl) and *Sst-Cre/tdTomato/Dnmt1 loxP*^{2} (KO) cells in E14.5 organotypic brain slices (350 µm coronal sections, time-period of imaging: 20 h). Analysis of the radial migration velocity and migrated path length is shown in **(a)** and **(b)**, respectively. **(c)** Migratory morphology of tdTomato^{+} cells investigated in organotypic brain slices (350 µm coronal sections). Nested two-way ANOVA ($p < 0.01$ **) with $N = 3$ embryos and $n = 4$ slices for Ctrl and KO animals, respectively, $n = 185$ cells for Ctr, and $n = 219$ KO cells. **(d-g)** siRNA-mediated depletion of *Dnmt1* in MGE-derived single cells *in vitro* (E14.5 + 1DIV). Scale bars: 10 µm. Quantification is shown in **(e-g)**. Unpaired two-tailed Student's *t*-test and unpaired Welch's *t*-test, $n = 60$ cells each for control siRNA and *Dnmt1* siRNA. siRNA: small-interfering RNA; LP: leading process, n.s.: not significant. **(h, i)** Exemplary microphotographs showing control siRNA-treated (upper panel) and *Dnmt1* KD (knockdown) MGE cells (E14.5 + 1DIV; lower panel) stained for Phalloidin647 and ERBB4. On the very right of each example, thermal-color-coded panels represent the respective fluorescent integrated density (IntDens) of ERBB4 (therm. (thermal) LUT). A dark blue color indicates 0, and a red color represents 5000 fluorescent units of the IntDens. Scale bars: 10 µm. **(i)** Quantification comparing the IntDens of ERBB4 fluorescence signals of Ctr and *Dnmt1* KD MGE cells. Unpaired two-tailed Student's *t*-test and unpaired Welch's *t*-test, $p < 0.001$ *** ($n = 88$ cells for control siRNA and 78 cells for *Dnmt1* siRNA, $N = 4$ experiments).

Data points depicted in (a) and (c) with the same symbol derive from one embryo. Error bars: +/- SEM of the mean (detailed information is provided by Supplementary Data 10).

Supplementary Figure S7:
***Dnmt1* deletion in *Sst-Cre/tdTomato* interneurons does not affect their cortical distribution in E16.5 and E18.5 embryos.**
(a) Microphotographs of tdTomato$^+$ cells in coronal sections (50 µm) of E16.5 *Som-Cre/tdTomato* (Ctrl) and *Som-Cre/tdTomato/Dnmt1 loxP^2* (KO) brains; scale bars: 500 µm. **(b)** Quantification of the proportional distribution of tdTomato$^+$ cells in the basal telencephalon and the cerebral cortex of E16.5 coronal sections (n = 11 for Ctrl and n = 15 for KO, N = 4 embryos per genotype). **(c)** Magnified microphotographs of the cortices of Ctrl and KO embryos from E16.5 coronal brain sections (50 µm). Scale bars: 100 µm. **(d)** Quantification of the proportional distribution of tdTomato$^+$ cells within the E16.5 cortical zones of Ctrl (n = 13 sections) and KO (n = 14 sections) embryos, analyzed in 50 µm coronal cryosections with N = 4 embryos per genotype. **(e)** Quantification of tdTomato-cell density normalized to the given area of the respective cortical zones (E16.5, n = 13 sections for Ctrl and n = 14 sections for KO, N = 4 embryos per genotype). **(f)** Microphotographs of tdTomato$^+$ interneurons in the cortex of coronally sectioned (50 µm) brains of E18.5 Ctrl and KO embryos; scale bars: 100 µm. **(g)** Quantification of the proportional distribution of tdTomato$^+$ cells within the E18.5 cortical zones normalized to the overall tdTomato-cell count within the cortex of Ctrl (n = 11 sections and KO n = 9 sections) embryos, analyzed in 50 µm coronal cryosections with N = 4 embryos per genotype. **(h)** Quantification of tdTomato-cell density normalized to the given area of the respective cortical zones at E18.5 (n = 11 sections for control and n = 9 sections for KO, N = 4 embryos per genotype). MZ: marginal zone, CP: cortical plate, IZ-VZ: intermediate zone to ventricular zone, n.s: not significant. TdTomato$^+$ cells: red and DAPI: blue. All quantifications were conducted using nested two-way ANOVA ($p < 0.05$ *, $p < 0.01$ **, $p < 0.001$ ***). Error bars: +/- SEM of the mean (detailed information is provided by Supplementary Data 10). Data points with the same symbol are from the same embryo.

Supplementary Figure S8: scRNA Seq data.
(a-e) Single-cell RNA sequencing (scRNA-seq) of E14.5 dorsal telencephalons **(a-c)** and E16.5 cortices **(e, f)** from C57BL/6J mice. UMAP illustrating cell clusters at E14.5 are depicted in **(a)**, and *Dnmt1* and *Sst* expression across these cluster are shown in **(b)** and **(c)**, respectively. **(d, e)** Panels show significant ligand-receptor pairs involved in communication from SST$^+$ cINs to cortical progenitors, filtered by differentially expressed genes (DEG) from single-cell RNA-sequencing data. Communication probability is represented by dot color, and p-value by dot size, with p-values being computed using a one-sided permutation test. Panel **(d)** shows the communication with different radial glia cell (RGC) populations at E16.5. Panel **(e)** illustrates communication with IPCs at E16.5.
(f-i) Single nuclear RNA (snRNA) sequencing of cortical cells prepared from E16.5 *Sst-Cre/tdTomato* and *Sst-Cre/tdTomato/Dnmt1 loxP^2* embryos (n = 2 brains from N = 2 mice per genotype). **(f)** UMAP depicting the cell clusters determined by snRNA sequencing across all samples. **(g)** Volcano plot collecting the DEGs in *Sst$^+$/Gad2$^+$* cells between E16.5 *Sst-Cre/tdTomato* and *Sst-Cre/tdTomato/Dnmt1 loxP^2* samples. **(h)** Dot plot depicting marker gene

expression in the different cell clusters. **(i)** UMAPs depicting the expression of relevant cluster-determining genes. Exact *p*-values and raw data are available via the hyperlinks listed in the Data Availability Statement.

Supplementary Figure S9: Spatial transcriptomic analysis using MERFISH
MERFISH was used to investigate the spatial distribution of different cell types in the developing mouse brain at E16.5 in *Sst-Cre/tdTomato* (Ctrl) and *Sst-Cre/tdTomato/Dnmt1 loxP*^{2} (KO) embryos. The 10 µm-thick coronal brain sections were processed and imaged with the MERSCOPE platform (Vizgen) and further analyzed on the single-cell level in R using Seurat. **(a)** Spatial distribution of identified clusters **(b)** Bar plot depicting the proportional distribution of cell types identified in cortical columns depicted in Figure 6. **(c)** Dot plot illustrating the expression of key marker genes across the identified cell clusters. **(d)** Distribution of select transcripts within control and knockout sections. Exact *p*-values and raw data are available via the hyperlinks listed in the Data Availability Statement.

Supplementary Figure S10: Phenotypic characterization of adult *Som-Cre/tdTomato* (Ctrl) and *Som-Cre/tdTomato/Dnmt1 loxP*^{2} (KO) cortices.
(a-d) TdTomato-expressing cells show immunoreactivity for neuropeptide Y and calretinin. Exemplary microphotographs of sagittal sections from a four-month-old *Sst-Cre/tdTomato* animal (Bregma 2.76). **(a** and **b)** Examples of immunohistochemical stainings using an anti-somatostatin antibody (Som, green) and an anti-neuropeptide Y antibody (NPY, magenta). TdTomato$^+$ cells are depicted in red. **(c** and **d)** Examples of immunohistochemical stainings using an anti-somatostatin antibody (SST, green) and an anti-calretinin antibody (Cal, magenta). TdTomato$^+$ cells are depicted in red. Scale bars in **(a)** and **(c)**: 100 µm. Scale bars in **(b)** and **(d)**: 20 µm (magnified views of sections outlined in **(a)** and **(c)**, respectively). **(e-h)** Immunostaining using an antibody directed against parvalbumin (PV) in 50 µm brain sections of six-month-old **(e, f)** *Sst-Cre/tdTomato* (control) and **(g, h)** *Sst-Cre/tdTomato/Dnmt1 loxP*^{2} (KO) mice. Exemplary microphotographs of immunostainings (tdTomato in red, PV in green, DAPI in blue) taken from the primary somatosensory cortex (S1) of coronal slices (Bregma 0.14, -0.22, and -0.34). Scale bars: 100 µm (left panels) and 20 µm (magnifications of marked selections). **(i)** Quantification of the proportion of tdTomato$^+$ cells expressing PV in *Sst-Cre/tdTomato* and *Sst-Cre/tdTomato/Dnmt1 loxP*^{2} mice across the cortical layers, normalized to the total amount of all detected tdTomato$^+$ cells. Nested two-way ANOVA with n = 11 slices for control and n = 10 for KO from N = 3 brains for both genotypes. $p < 0.05$ *, $p < 0.01$ **, $p < 0.001$ ***, n.s.: not significant. Error bars: +/- SEM of the mean (detailed information is provided by Supplementary Data 10). Data points sharing the same symbol represent one mouse

Supplementary Figure S11:
Neuropixels recordings in adult *Sst-Cre/tdTomato/Dnmt1-loxP*^{2} (KO) and *Sst-Cre/tdTomato* (Ctrl) mice
(a, b) Histological localization of Neuropixels probes in S1 (left) and S1 (right) of *Sst-Cre/tdTomato* (Ctrl, **a**) and *Sst-Cre/tdTomato/Dnmt1-loxP*^{2} (KO, **b**) mice. Each electrode was coated with fluorescent DiD for post-hoc verification. After recordings, brains were cut (50 µm

slices) and stained with DAPI (blue). Based on anatomical landmarks, slices were aligned to the Allen CCF to verify correct probe positions. **(c-h)** Adult KO mice show functional abnormalities. **(c)** Left and center: Average S1 LFP across cortical depth, following a 20-ms air puff stimulus (white line) to the whisker pad. Blue colors indicate negative, and yellow colors positive LFP responses. Right: Across all layers, LFP responses were stronger and more temporally precise in Ctrl (grey) compared to KO (orange). **(d)** Quantification of spiking responses to tactile stimulation for neurons in the supra- and infragranular S1 layers (see also Fig. 7i). Early spiking responses within the first 30 ms after stimulus onset were significantly reduced in KO mice, especially in the supragranular layers (early $response_{control}$ = 11.99 ± 0.86 hz, early $response_{KO}$ = 4.58 ± 0.68 hz, $p < 8.5^{-35}$, $n_{control}$ = 183 neurons from 2 mice, n_{KO}= 60 neurons from 2 mice). Supragranular tactile responses were also longer lasting in KO mice, with significantly increased late sensory responses from 100 to 300 ms (late $response_{control}$ = 2.09 ± 0.48 hz, late $response_{KO}$ = 5.92 ± 1.35 hz, p = 0. 0003). **(e)** Optogenetic stimulation of SST^+ cINs in V1 and S1 with 20-ms-long flashes of blue light (10 mW, 488 nm), to isolate SST^+ cIN activity. The action potential waveform of light-responsive neurons was largely similar between genotypes. Spike width was also not significantly different (spike $width_{control}$ = 1.04 ± 0.01 ms, spike $width_{KO}$ = 1.05 ± 0.03 ms, p = 0. 8271, $n_{control}$ = 73 neurons, 2 mice, n_{KO}= 42 neurons, 2 mice). **(f)** Spontaneous firing rate of the same SST^+ cINs as in (**c**) for Ctrl and KO (firing $rate_{control}$ = 2.56 ± 0.7 Hz, firing $rate_{KO}$ = 1.79 ± 1.12 Hz, p = 0. 2958). **(g)** Left: Peristimulus time histogram for S1 and V1 neurons that were suppressed by optogenetic stimulation of SST^+ cINs in the same area for Ctrl (gray) and KO mice (orange). Optogenetic stimulus started at time 0, ramped up for 0.5 seconds, and remained on for 1 second. Right: Optogenetic stimulation of SST^+ cINs equally suppressed other cortical neurons in both groups (firing rate $change_{control}$ = -4.69 ± 0.15 Hz, firing rate $change_{KO}$ = -4.65 ± 0.20 Hz, p = 0. 8352, $n_{control}$ = 266 neurons from 2 mice, n_{KO}= 123 neurons from 2 mice). **(h)** Spontaneous firing rate of all cortical neurons across depth. The spontaneous firing rate of non-SST^+ cINs was significantly reduced in KO mice, especially in the supragranular layers (firing $rate_{control}$ = 1.25 ± 0.48 Hz, firing $rate_{KO}$ = 0.43 ± 0.52 Hz, p = 0.0015, $n_{control}$ = 262 neurons from 2 mice, n_{KO}= 93 neurons from 2 mice). Significance for panels d, e, f, g was based on a Wilcoxon *rank-sum* test, values are mean ± SEM. Shading in panels (c) and (g) shows the SEM. Exact *p*-values and raw data are available via the hyperlinks listed in the Data Availability Statement.

Supplementary Figure S12:

Behavioral analyses of adult *Sst-Cre/tdTomato* and *Sst-Cre/tdTomato/Dnmt1 loxP*^{2} mice.

(**a-d**) Evidence accumulation test (unisensory (visual or tactile) or multisensory (visuotactile)). Exact *p*-values and raw data are available via the hyperlinks listed in the Data Availability Statement. (**a**) Learning curves from *Som-Cre/tdTomato* (Ctrl) and *SomCre/tdTomato/Dnmt1 loxP*^{2} (KO) mice across display the correctly performed trials per session in % (successful learning: sustained performance > 75% (red dashed lines)). Linear mixed effects model (LME), n = 18 binned sessions from N = 6 mice per modality, p_{visual} = 0.89, $p_{tactile}$ = 0.64, $p_{multisensory}$ = 0.29). (**b**) Multisensory enhancement (LME, n = 98 sessions from N= 6 mice, p = 0.95). (**c, d**) Psychometric curves across all modalities for Ctrl and KO mice across difficulty levels (interhemispheric stimulus difference).

Both side-specific (**c**) and side-independent (**d**) performances (LME, n = 18 performance values from N = 6 mice over 3 difficulties, p_{visual} = 0.78, $p_{tactile}$ = 0.33, $p_{multisensory}$ = 0.26). (**e-g**) Learning and visual navigation were tested in a Morris water maze (MWM) over six days (N = 7 Ctrl, N = 6 KO). **(e)** Scheme of the MWM task. **(f)** Successful escapes are shown as the percentage of trials in which the animals found the platform (binomial test: p_{visual} = 0.32). **(g)** Covered distances per session (two-way ANOVA: $p_{Genotype}$ = 0.238, $p_{Session}$ = 3.4^{-10}, $p_{Genotype\ x\ Session}$ = 0.889). Raw data of (f) and (g) are listed in Supplementary Table 10. **(h, i)** Chemoconvulsant susceptibility was assessed using PTZ-induced seizures scored according to Racine's scale (see methods). Average time point and applied dosage of PTZ for provoking the first occurrence of different epileptic seizure forms. N = 10 for both *Dnmt1* genotypes (severity level 4: N = 10 Ctrl and N = 9 KO mice; severity level 5: N = 10 Ctrl and KO mice each; severity level 6: N= 4 Ctrl and N = 5 KO mice*;* severity level 7: N= 9 Ctrl and N = 7 KO mice **(h)** Time to first seizure occurrence [0 min]). **(i)** Cumulated PTZ dosages (one injection: 10 mg PTZ/kg body weight) to elicit the initial occurrence of each respective Racine's score severity level. Unpaired, two-tailed Student's *t*-test and additional unpaired Welch's *t*-test; n.s.: not significant. Error bars: +/- SEM of the mean (more detailed information is provided by Supplementary Data 10).

3. Supplementary Tables and Legends

Supplementary Table S1*.*

The number of contacts for $A^{\wedge 2}$ of interface area (calculated by dr-sasa web server[2]) of selected protein/DNA complexes with affinities in the nM range. Affinity data from the ProNAB database[3]. All selected structures in solution (either by NMR or by the end of our three MD simulations), except for the DNMT1/UMDNA/SAH complex X-ray structure (PDB ID: 3PTA[4]).

PDB ID	Kd (nM)	Number of contacts with a cutoff at 4 Å	Protein/DNA interfacial area ($A^{\wedge 2}$)	Ratio
1IV6[5]	200.0	194	961	0.20
1J5N[6]	10.0	296	1740	0.20
1MSE[7]	11.2	275	1377	0.17
1GCC[8]	4.1	164	800	0.21
3PTA[4]	2.3	97	761	0.13
MD	2.3	184±17	1102±54	0.17

Supplementary Table S2.

Interaction list between the catalytic domain and the CpG site in DNMT1/HMDNA/SAH complex was determined by Cryo-EM (PDB ID: 7XI9[9]) and by the end of our three MD simulations (residues within 4 Å).

	7XI9[9]	Replica1	Replica2	Replica3
**LYS1275**	×	√	×	√
**ARG1276**	×	×	√	√
**MET1232**	√	×	×	×
**ASN1233**	√	×	×	×
**ARG1234**	√	√	√	√
**ASN1236**	√	√	×	√

Supplementary Table S3

The atomistic partial charges of SAM (**Supplementary Figure 1**) in this study.

Atom	Charge	Atom	Charg e	Atom	Charge	Atom	Charge	Atom	Charge
N1	-0.486	O11	-0.382	C21	0.602	H31	0.400	H41	0.122
C2	0.024	C12	0.159	C22	-0.062	H32	0.245	H42	0.159
C3	0.661	O13	-0.650	C23	0.682	H33	0.245	H43	0.0889
C4	-0.689	C14	0.152	N24	-0.807	H34	0.164	H44	0.096
C5	0.032	O15	-0.618	N25	-0.546	H35	0.164	H45	0.112
C6	-0.249	C16	0.139	C26	0.154	H36	0.193	H46	0.057
C7	0.337	O17	-0.689	N27	-0.186	H37	0.193	H47	0.134
C8	-0.355	N18	-0.770	H28	0.451	H38	0.193	H48	0.347
C9	-0.450	C19	0.548	H29	0.424	H39	0.067	H49	0.347
C10	0.219	N20	-0.785	H30	0.400	H40	0.067	H50	0.347

Supplementary Table S4.

Parameters of Zn(II) ions (**Supplementary Figure S1**) in this study.

	Type	LJ Radius (Å)	LJ Depth (kcal/mol)	Charge (e)	GB Radius (Å)	GB Screen
Zn1	Cys1476-Cys1478-Cys1485-His1502	1.3730	0.0118	0.6157	1.5000	0.8000

Zn2	Cys653-Cys656-Cys659-Cys691	1.3730	0.0118	0.8827	1.5000	0.8000
Zn3	His793-Cys820-Cys893-Cys896	1.3730	0.0118	0.6757	1.5000	0.8000
Zn4	Cys664-Cys667-Cys670-Cys686	1.3730	0.0118	0.6654	1.5000	0.8000

Supplementary Table S5.

Simulated system in this study.

System	Charge of complex	Number of K^+ ions	Number of Na^+ ions	Number of Cl^- ions	Number of H_2O molecules
DNMT1/UMDNA/SAM	-21	157	14	150	39.570

Supplementary Table S6.

Additional Information Microscopy: Settings used for imaging with the Leica DMi8.

**Channel**	**Wavelength [nm]**	**Max. LED intensity [%]**	**Max. illumination time [ms]**
Cy5	650	50	500
DAPI	395	20	200
FITC	488	50	500
TRITC	544/545	50	500

Supplementary Table S7

Additional Information Microscopy: LED settings used for pair cell imaging with Leica DMi8 HC PL FLUOTAR L 40x/0.60 DRY.

**Channel**	**Wavelength [nm]**	**Max. LED intensity [%]**	**Max. illumination time [ms]**
Cy5	650	60	500

DAPI	395	23	300
FITC	488	60	300
TRITC	544/545	60	500

4. Supplementary Methods

Model Construction

Our structure model of the DNMT1/UMDNA/SAM complex was based on the X-ray structures of DNMT1/UMDNA/SAH (PDB ID: 3PTA[4]) and of DNMT1/SAM (PDB ID: 3AV6) [10]. The missing loops in DNMT1 were modeled using the SWISS-MODEL web server[11]. The protonation states of DNMT1's ASP, GLU, ARG, LYS, and HIS residues, as well as those of the UMDNA strand (5'-TpCpCpCpGpTpGpApGpCpCpTpCpCpGpCpApGpGp-3'), were determined using the H++ web server[12] and tLEaP based on the Amber OL21 force field[13] assuming a pH 7.4 to mimic physiological conditions.

The force fields for the protein, methylated cytosine, UMDNA, water, and ions were AMBER ff19SB force fields[14], parameter set from Lankas et al.[15], Amber OL21 force field[13], OPC[16], and Åqvist potential[17], respectively. Those of SAM (**Supplementary Fig. S1l, and Supplementary Table S3**) and the Zn coordination polyhedron (**Supplementary Fig S1m, and Supplementary Table S4**) were consistent with the used protein force field: The charges were derived from the geometry-optimized structure at the B3LYP level of theory (with Grimme's dispersion correction[18]). The basis sets were 6-311G(d,p) and 6-31G(d), respectively. We used the restrained electrostatic potential (RESP) fitting method[19], following the Merz-Kollman (MK) scheme[20]. The van der Waals parameters were taken from the previous work of Li et al.[21] . The bonded parameters of the Zn coordination polyhedron were calculated at the same level of theory and obtained by the Seminario method[22]. Those for SAM were from GAFF2[23]. All the quantum mechanics (QM) calculations are performed by Gaussian09[24].

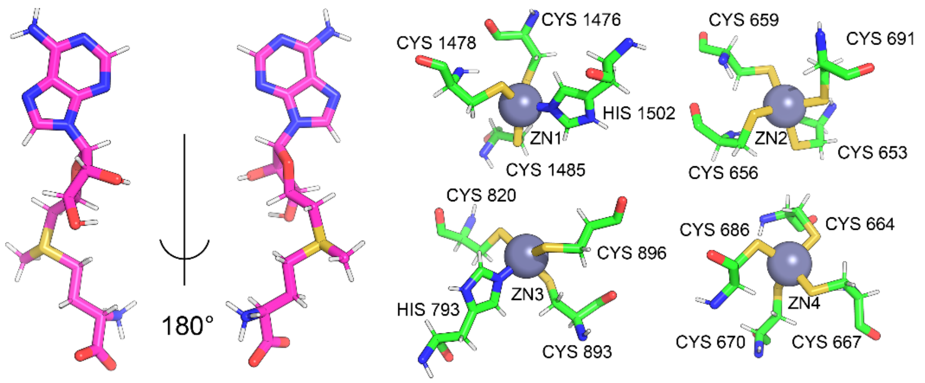

Supplementary Methods Figure 1: Three-dimensional schematic diagram of SAM (left) and Zn coordination polyhedron (right). The parameters of SAM and Zn(II) ions are provided in **Supplementary Tables S3 and S4**.

Details on generating transgenice mice:
The generation of control animals was conducted by crossing a *Sst*$^{tm2.l(cre)Zjh}$/J strain (RRID:IMSR_JAX:013044, Jackson Laboratory, Bar Harbour, U.S.A.) with a t*dTomato* reporter mouse model (*B6.CgGt(ROSA)26Sor*$^{tml\ 4(\ CAG\text{-}tdTomato\ )Hze}$*,* obtained from Christian Hübner, University Hospital Jena, Germany). An *internal ribosomal entry site* (*IRES*), a *Cre-recombinase* sequence, a *polyA* sequence, and a *frt*-flanked neo cassette were inserted into the 3' untranslated region (UTR) of the *Somatostatin (Sst)* locus on chromosome 16, limiting the respective *Cre*-expression to *Sst*-positive cells. The t*dTomato* reporter strain harbors the *tdTomato* sequence combined with a *loxP*-flanked stop cassette in the *Rosa26* locus. Expression of *Cre-recombinase* dependent on *Sst*-promoter activity resulted in the deletion of the *loxP*-flanked stop cassette during recombination and an expression of *tdTomato* in *Sst-Cre*-positive cells. *Jackson Laboratory* recommends creating and breeding *Sst-Cre* heterozygous mice (jax.org/Strain/013044), which received the *Cre*-allele from the maternal side. This strategy avoids potential instability of the Cre-recombinase activity during paternal germline recombination[25]. To generate the triple transgenic conditional KO-model *Sst-Cre*$^{+/-}$*/tdTomato* females were bred with male individuals derived from crossing *tdTomato* mice with a *Dnmt1 loxP*^{2} strain (*B6; 129Sv-Dnmt1tm2Jae/J,* obtained from Rudolf Jaenisch, Whitehead Institute for Biomedical Research Boston, U.S.A.) in which exon 4 and 5 of the *Dnmt1* gene are loxP-flanked, resulting in a null allele of these loci and a subsequent DNMT1 deficiency[26]. Corresponding programs and primers used for genotyping can be found in the supplementary information. An illustration of control- and KO mice is shown in Supplementary Figure S2a.
Housing and breeding took with the permission for animal husbandry operations (§11 Animal Protection Act). All mice were housed at 20-24 °C with a relative air humidity of 45-65 % and under constant light-dark cycle of 12 h (less than 200 Lux at a height of 1 m). Food and water was available *ad libitum* in standardized and individually ventilated IVC cages (Tecniplast, Germany) with additional activity and nest building material. All general procedures (housing, breeding) were carried out at the Institute of Zoology (Biology II, RWTH Aachen) and at the Center for Experimental Models and Transgenic Service (CEMT), University Medical Center Freiburg, in accordance with European Directive 2010/63/EU (ETS123), FELASA standards, and the German GV-SOLAS guidelines, with approval for animal husbandry according to §11 Animal Protection Act (TierSchG). For the procedures conducted at the RWTH Aachen, organ removal was performed under license 40168.A4, approved by the Institute for Laboratory Animal Research, University Hospital Aachen. Embryo isolation followed protocol 81-02.04.2019.A311, authorized by the LANUV (North Rhine-Westphalia). Behavioral experiments, including PTZ, Neuropixels recordings, Morris Water Maze, and (multi)sensory discrimination tasks, were conducted under protocol 81-02.04.2020.A175 and complied with the same European and national regulations. Procedures conducted on C57BL6/J mice at the University Medical Center Freiburg were approved by the Regierungspräsidium Freiburg under the licenses G19/125 and G21/082 and in accordance with §4 TierSchG.

DNA extraction and genotyping

Ear biopsies as well as embryonic tissue and tail biopsies from mice killed in experiments were incubated in an alkaline lysis buffer (25 mM NaOH, 0.2 mM EDTA, volume adapted to tissue size) for 90 minutes at 96°C and 350 rpm using a ThermoMixer® (Eppendorf AG, Germany). Subsequently, samples were cooled down on ice and neutralized using the same volume of 40 mM Tris-HCl. Next, amplification of genomic DNA was performed in a T100 thermal cycler (BioRad, U.S.A.) with the help of the corresponding primer sequences and PCR programs listed below. For this, 1 µL of isolated DNA was incubated together with 19 µL of PCR master mix containing 2x FastGene™ Optima reaction mix (Nippon Genetics, Japan), nuclease-free H_2O, and respective primers. Product size determination via electrophoresis was conducted using a 2% agarose gel (2% agarose/1x Tris-acetate-EDTA (TAE) buffer) containing MidoriGreen™ (Nippon Genetics, Japan). Additionally, a 1 kb DNA ladder was applied according to the manufacturer's guidelines (GeneRuler™, Thermo Fisher Scientific, U.S.A.). Gel electrophoresis was performed applying 190 V using a PowerPac power supply (BioRad, U.S.A.). Final detection of genotyping results was conducted via GelDoc™XR imaging system (BioRad, U.S.A.) and the corresponding software Image Lab (BioRad, U.S.A.).

Primer sequences and polymerase chain reaction (PCR) programs used for genotyping

Somatostatin-Cre

- Universal reverse primer: 5'-GGG CCA GGA GTT AAG GAA GA-3'
- Wildtype forward primer: 5'-TCT GAA AGA CTT GCG TTT GG-3'
- Mutant forward primer: 5'-TGG TTT GTC CAA ACT CAT CAA-3'

TdTomato

- Wildtype forward primer: 5'-AAG GGA GCT GCA GTG GAG TA-3'
- Wildtype reverse primer: 5'-CCG AAA ATC TGT GGG AAG TC-3'
- Mutant forward primer: 5'-GGC ATT AAA GCA GCG TAT CC-3'
- Mutant reverse primer: 5'-CTG TTC CTG TAC GGC ATG G-3'

Dnmt1 loxP[2]

- Forward primer: 5'-GGG CCA GTT GTG TGA CTT GG-3'
- Reverse primer: 5'-CCT GGG CCT GGA TCT TGG GGA-3'

	94 °C, 5 min
⌈	94 °C, 30 s
9x	65 °C, 30 s
⌊	72 °C, 30 s
⌈	94 °C, 30 s
40x	60 °C, 30 s
⌊	72 °C, 30 s
	72 °C, 5 min
	4 °C, ∞

	95 °C, 3 min
⌈	95 °C, 15 s
9x	61 °C, 20 s
⌊	72 °C, 30 s
	72 °C, 3 min
	12 °C, ∞

	95 °C, 3 min
⌈	95 °C, 30 s
37x	58 °C, 25 s
⌊	72 °C, 30 s
	72 °C, 2 min
	4 °C, ∞

Chromatin immunoprecipitation (ChIP) and sequencing

Cerebellar granule (CB) cells[27] were cultured and treated as previously described[28]. The used CB cell line was first described in Fossale et al. (2004; https://link.springer.com/article/10.1186/1471-2202-5-57) and was obtained by Dr. Mukhran Khundadze (University Hospital Jena, Germany). DNMT1-interacting chromatin was immunoprecipitated via native chromatin immunoprecipitation (ChIP)[28]. The sequencing libraries were prepared using the NEBNext® Ultra II DNA Library Prep kit (#E7410S, New England Biolabs, U.S.A.) as per manufacturer's instructions. The input controls were pooled across samples in equimolar proportions. The libraries were amplified via PCR on a T100 Thermal Cycler (Bio-Rad, U.S.A.) with the following conditions: initial denaturation at 98°C for 30 s, 15 cycles of denaturation at 98°C for 10 s and annealing/extension at 65°C for 75 s, followed by a final extension at 65°C for 5 min. To account for the laddering caused by the enzymatic digestion, the libraries were size selected on a 2% (v/v) agarose gel and purified using the QIAquick Gel Extraction Kit (#28704, Qiagen, Germany). The libraries were sequenced on the NextSeq platform using 75 bp single-end reads at the IZKF Genomics Facility (University Hospital Aachen).

FASTQ files generated from the ChIP-seq experiment were processed using the nf-core/chipseq pipeline (version 2.0.0) with the mm10 reference genome[29]. Differential peak calling between the samples was performed using THOR with default settings[30]. The identified peaks with -log10*p* > 1.4 in the control samples are listed in Supplementary Table 4. Sequences within a 500 bp window surrounding these peaks were analyzed for motif detection using MEME-ChIP[31].

Details on microscopes and applied settings

Detection of EOMES (E14.5, E16.5), TBR1 (E14.5, E16.5), SOX2 (E14.5), and DNMT1 immunostainings in embryonic sections (E14.5), microscopic imaging was conducted in tile scans, 2x2 binning, and with a HC PL FLUOTAR L20x/0.40 objective in a confocal-like Leica DMi8 fluorescent microscope in combination with a THUNDER® imager unit (Leica, Germany) and the corresponding software *LASX* (Leica, Germany). TdTomato signals were detected with the TRITC channel, and DNMT1, EOMES, TBR1, and SOX2 with the Cy5 channel (DNMT1, EOMES, and TBR1 in false color green). DAPI was captured with the settings for the DAPI channel (Supplementary Table S6). For post-processing, the *LASX* software with the "*Mosaic Merge*"- and "*Thunder Lightning (Large Volume)*" tools were used. A maximum intensity projection of the merged tile scans was done with the *Fiji* software[105] and *LASX*. DNMT1^{+} cell detection and quantification were performed using QuPath, applying threshold-based segmentation on fluorescence intensity to identify labeled cells. For the detection of TBR1 and EOMES in E18.5 sections a Keyence BZ-X810 fluorescence microscope (Keyence Corporation, Japan) with a BZ-PA10 10x/ 0.45 objective was used, in combination corresponding Keyence BZ-X800 Software (z-stack: 16 levels à 2 μm) and tile scan imaging with *High Resolution* settings in *Low Photobleach Mode*). Post-processing z-levels in combination with the *Full Focus* setting, and afterwards all projected tile scans were stitched with the BZ-X800 analyzer program. Corresponding Keyence filter cubes were used to excite DAPI (395 nm, 3.3 ms 100 % excitation), tdTomato (TRITC 545 nm, 200 ms, 100% excitation),

and EOMES and TBR1 with Cy5 (620 nm, 200 ms, 100 % excitation). EOMES and TBR1 were depicted as false color green.

All adult sections, except for PV-immunohistochemical labelings, were captured with the Leica DMi8 microscope with THUNDER® module. Respective microphotographs were either captured with a HC PL FLUOTAR L20x/0.40 or HC PL APO 40x/1.30, the latter together with oil immersion (DNMT1). DNMT1^{+} cell detection and quantification were performed using QuPath, applying threshold-based segmentation on fluorescence intensity to identify labeled cells. PV-associated fluorescent signals were detected using the already mentioned Keyence BZ-X810 fluorescence microscope with a BZ-PA10 10x/0.45 objective and *High Resolution* settings in *Low Photobleach Mode* in the corresponding Keyence BZ-X800 software in several sections of each slice. Merging of the resulting tile scans was performed either by the Keyence Analyzer software or the Grid/Collection Stitching Plugin in Fiji. A final maximum intensity projection was done by Fiji. The following channels were used for the respective antibody-associated fluorophores. TdTomato: TRITC; NPY, calretinin, and DNMT1: Cy5; SST and PV: FITC (Supplementary Table S6).

Morphology- and ERBB4-associated detection of Phalloidin645 and Cy3 signals in dissociated single cells was also conducted via the already mentioned Leica DMi8 using a HC PL APO 40x/1.30 in combination with oil immersion. Cells were captured via z-stacks with 2-5 μm step size and post-processed using the THUNDER ® Lightning Instant Computational Clearing tool (ICC) of the *LASX* software. Phalloidin645 was detected using Cy5 parameters, ERBB4-antibody-coupled Cy3 fluorophores via TRITC channel settings, and DAPI via DAPI-channel (Supplementary Table S6). For pair-cell assay analyses cortical cells were captured with Objective HC PL FLUOTAR 40x/0.60 CORR PH2 air lens. Associated fluorophores were detected as follows: NES: Cy5, TUBB3: FITC, EOMES: FITC (in false color yellow), DAPI: DAPI channel (Supplementary Table S6).

5. Supplementary References:

1. Singh, M., Saxena, S. & Mohan, K. N. DNMT1 downregulation as well as its overexpression distinctly affect mostly overlapping genes implicated in schizophrenia, autism spectrum, epilepsy, and bipolar disorders. *Front. Mol. Neurosci.* **16**, 1275697 (2023).
2. Ribeiro, J., Ríos-Vera, C., Melo, F. & Schüller, A. Calculation of accurate interatomic contact surface areas for the quantitative analysis of non-bonded molecular interactions. *Bioinformatics* **35**, 3499–3501 (2019).
3. Harini, K., Srivastava, A., Kulandaisamy, A. & Gromiha, M. M. ProNAB: database for binding affinities of protein–nucleic acid complexes and their mutants. *Nucleic Acids Research* **50**, D1528–D1534 (2022).
4. Song, J., Rechkoblit, O., Bestor, T. H. & Patel, D. J. Structure of DNMT1-DNA Complex Reveals a Role for Autoinhibition in Maintenance DNA Methylation. *Science* **331**, 1036–1040 (2011).
5. Nishikawa, T. *et al.* Solution Structure of a Telomeric DNA Complex of Human TRF1. *Structure* **9**, 1237–1251 (2001).
6. Masse, J. E. *et al.* The S.cerevisiae Architectural HMGB Protein NHP6A Complexed with DNA: DNA and Protein Conformational Changes upon Binding. *Journal of Molecular Biology* **323**, 263–284 (2002).

7. Ogata, K. *et al.* Solution structure of a specific DNA complex of the Myb DNA-binding domain with cooperative recognition helices. *Cell* **79**, 639–648 (1994).
8. Allen, M. D., Yamasaki, K., Ohme-Takagi, M., Tateno, M. & Suzuki, M. A novel mode of DNA recognition by a β-sheet revealed by the solution structure of the GCC-box binding domain in complex with DNA. *EMBO J* **17**, 5484–5496 (1998).
9. Kikuchi, A. *et al.* Structural basis for activation of DNMT1. *Nat Commun* **13**, 7130 (2022).
10. Takeshita, K. *et al.* Structural insight into maintenance methylation by mouse DNA methyltransferase 1 (Dnmt1). *Proc. Natl. Acad. Sci. U.S.A.* **108**, 9055–9059 (2011).
11. Waterhouse, A. *et al.* SWISS-MODEL: homology modelling of protein structures and complexes. *Nucleic Acids Research* **46**, W296–W303 (2018).
12. Anandakrishnan, R., Aguilar, B. & Onufriev, A. V. H++ 3.0: automating pK prediction and the preparation of biomolecular structures for atomistic molecular modeling and simulations. *Nucleic Acids Research* **40**, W537–W541 (2012).
13. Zgarbová, M., Šponer, J. & Jurečka, P. Z-DNA as a Touchstone for Additive Empirical Force Fields and a Refinement of the Alpha/Gamma DNA Torsions for AMBER. *J. Chem. Theory Comput.* **17**, 6292–6301 (2021).
14. Tian, C. *et al.* ff19SB: Amino-Acid-Specific Protein Backbone Parameters Trained against Quantum Mechanics Energy Surfaces in Solution. *J. Chem. Theory Comput.* **16**, 528–552 (2020).
15. Lankaš, F. *et al.* Critical Effect of the N2 Amino Group on Structure, Dynamics, and Elasticity of DNA Polypurine Tracts. *Biophysical Journal* **82**, 2592–2609 (2002).
16. Izadi, S., Anandakrishnan, R. & Onufriev, A. V. Building Water Models: A Different Approach. *J. Phys. Chem. Lett.* **5**, 3863–3871 (2014).
17. Åqvist, J. Ion-water interaction potentials derived from free energy perturbation simulations. *J. Phys. Chem.* **94**, 8021–8024 (1990).
18. Grimme, S., Antony, J., Ehrlich, S. & Krieg, H. A consistent and accurate *ab initio* parametrization of density functional dispersion correction (DFT-D) for the 94 elements H-Pu. *The Journal of Chemical Physics* **132**, 154104 (2010).
19. Bayly, C. I., Cieplak, P., Cornell, W. & Kollman, P. A. A well-behaved electrostatic potential based method using charge restraints for deriving atomic charges: the RESP model. *J. Phys. Chem.* **97**, 10269–10280 (1993).
20. Singh, U. C. & Kollman, P. A. An approach to computing electrostatic charges for molecules. *J Comput Chem* **5**, 129–145 (1984).
21. Li, P., Roberts, B. P., Chakravorty, D. K. & Merz, K. M. Rational Design of Particle Mesh Ewald Compatible Lennard-Jones Parameters for +2 Metal Cations in Explicit Solvent. *J. Chem. Theory Comput.* **9**, 2733–2748 (2013).
22. Seminario, J. M. Calculation of intramolecular force fields from second-derivative tensors. *Int. J. Quantum Chem.* **60**, 1271–1277 (1996).
23. Wang, J., Wolf, R. M., Caldwell, J. W., Kollman, P. A. & Case, D. A. Development and testing of a general amber force field. *J Comput Chem* **25**, 1157–1174 (2004).
24. M. J. Frisch, G. W. Trucks, H. B. Schlegel, G. E. Scuseria, M. A. Robb, J. R. Cheeseman, G. Scalmani, V. Barone, B. Mennucci, G. A. Petersson, H. Nakatsuji, M. Caricato, X. Li, H. P. Hratchian, A. F. Izmaylov, J. Bloino, G. Zheng, J. L. Sonnenberg, M. Hada, M. Ehara, K. Toyota, R. Fukuda, J. Hasegawa, M. Ishida, T. Nakajima, Y. Honda, O. Kitao, H. Nakai, T. Vreven, J. A. Montgomery, Jr., J. E. Peralta, F. Ogliaro, M. Bearpark, J. J. Heyd, E. Brothers, K. N. Kudin, V. N. Staroverov, R. Kobayashi, J. Normand, K. Raghavachari, A. Rendell, J. C. Burant, S. S. Iyengar, J. Tomasi, M. Cossi, N. Rega, J. M. Millam, M. Klene, J. E. Knox, J. B. Cross, V. Bakken, C. Adamo, J. Jaramillo, R. Gomperts, R. E. Stratmann, O. Yazyev, A. J. Austin, R. Cammi, C. Pomelli, J. W. Ochterski, R. L. Martin, K. Morokuma, V. G. Zakrzewski, G. A. Voth, P. Salvador, J. J. Dannenberg, S. Dapprich, A. D. Daniels, O. Farkas, J. B. Foresman, J. V. Ortiz, J. Cioslowski, and D. J. Fox,. Gaussian 09, Revision A.02. (2009).
25. Madisen, L. *et al.* A robust and high-throughput Cre reporting and characterization system for the whole mouse brain. *Nat Neurosci* **13**, 133–40 (2010).

26. Jackson-Grusby, L. *et al.* Loss of genomic methylation causes p53-dependent apoptosis and epigenetic deregulation. *Nat Genet* **27**, 31–39 (2001).
27. Fossale, E. *et al.* Membrane trafficking and mitochondrial abnormalities precede subunit c deposition in a cerebellar cell model of juvenile neuronal ceroid lipofuscinosis. *BMC Neurosci* **5**, 57 (2004).
28. Yildiz, C. B. *et al.* EphrinA5 regulates cell motility by modulating Snhg15/DNA triplex-dependent targeting of DNMT1 to the Ncam1 promoter. *Epigenetics & Chromatin* **16**, 42 (2023).
29. Ewels, P. A. *et al.* The nf-core framework for community-curated bioinformatics pipelines. *Nat Biotechnol* **38**, 276–278 (2020).
30. Allhoff, M., Seré, K., F Pires, J., Zenke, M. & G Costa, I. Differential peak calling of ChIP-seq signals with replicates with THOR. *Nucleic Acids Res* **44**, e153 (2016).
31. Machanick, P. & Bailey, T. L. MEME-ChIP: motif analysis of large DNA datasets. *Bioinformatics* **27**, 1696–1697 (2011).
