# Supplementary material for: DNMT1-mediated regulation of somatostatin-positive interneuron migration impacts cortical architecture and function
Source: Nat Commun. 2025 Jul 24;16:6834. doi: 10.1038/s41467-025-62114-0 (PMC12290092; doi:10.1038/s41467-025-62114-0)
Supplement: Supplementary file 2 — Description of Additional Supplementary Files [file 41467_2025_62114_MOESM2_ESM.pdf]

Description of Additional Supplementary Files

Title: Supplementary Data 1. (Supplementary Data 1-RNA Seq FACS SST-KO and ctrl E14.xlsx)
Description:
Comparison of differential gene expression in tdTomato-positive interneurons of the basal telencephalon of E14.5 *Sst-Cre/tdTomato/Dnmt1 loxP*^{2} and *Sst-Cre/tdTomato* control mice as revealed by RNA sequencing. Differentially expressed genes (DEG) are listed in sheet 1-1, genes with reduced expression in *Sst-Cre/tdTomato/Dnmt1 loxP*^{2} are listed in sheet 1-2, while genes found upregulated in *Sst-Cre/tdTomato/Dnmt1 loxP*^{2} are listed in sheet 1-3.

Title: Supplementary Data 2. (Supplementary Data 2-DMRs FACS SST-KO and ctrl E14.xlsx)
Description:
Differentially methylated regions in tdTomato-positive interneurons of the basal telencephalon of E14.5 *Sst-Cre/tdTomato/Dnmt1 loxP*^{2} and *Sst-Cre/tdTomato* control mice as revealed by methyl-sequencing. Differentially methylated regions (DMRs) are listed in sheet 1-1. Negative values indicate increased methylation in KO.

Title: Supplementary Data 3. (Supplementary Data 3-Overlap DEG-DMG SST-Dre-DNMT1 E14.5Venn_Data.xlsx)
Description:
Collection of genes that were both differentially expressed and differentially methylated in tdTomato-positive interneurons of the basal telencephalon of E14.5 *Sst-Cre/tdTomato/Dnmt1 loxP*^{2} and/or *Sst-Cre/tdTomato* control mice as revealed by RNA and methyl-sequencing, respectively, building the basis of the Venn diagrams depicted in Figure 1. DEG_up refers to upregulated genes in *Sst-Cre/tdTomato/Dnmt1 loxP*^{2} samples.

Title: Supplementary Data 4. (Supplementary Data 4 – ChIP seq THOR peaks p1.4.xlsx)
Description:
DNMT1 ChIP-seq peaks in immortalized cerebellar granule (CB) cells. The table lists the chromosomal locations of DNMT1-interacting chromatin segments with a *p* value cut-off of 1.4 (-log10) as well as the corresponding genes on the coding and the template strand, marked by (+) and (-), respectively. The peaks were generated by the THOR algorithm with the default parameters[1]. Genes differentially expressed after Dnmt1 Knockout were tested for the presence of the identified Motif using the AME algorithm supplied with the Motif and the transcription start sites (+-3kb).

Title: Supplementary Data 5. (Supplementary Data 5-GO_enrichment_DEGup_and_DMR.csv)
Description:
Gene ontology (GO) – biological process analysis of genes with increased expression and reduced methylation in E14.5 *Sst-Cre/tdTomato/Dnmt1 loxP*^{2} samples compared to *Sst-Cre/tdTomato* cells.

Title: Supplementary Data 6. (Supplementary Data 6-GO_BiologicalP_DEG_up.csv)
Description:
Gene ontology (GO) – biological process analysis (ShinyGO 0.82; https://bioinformatics.sdstate.edu/go) of all genes with increased expression in E14.5 *Sst-Cre/tdTomato/Dnmt1 loxP*^{2} samples compared to *Sst-Cre/tdTomato* cells.

Title: Supplementary Data 7. (Supplementary Data 7-Overlap DEG SST-Cre DNMT1 KO withDNMT1 OE)
Description:
Collection of genes that were differentially expressed in both E14.5 *Sst-Cre/tdTomato/Dnmt1 loxP*^{2} samples compared to *Sst-Cre/tdTomato* cells, as well as in *Dnmt1* overexpressing neurons

transdifferentiated from murine ESCs compared to their controls. This table also provides the basis for the Venn Diagram depicted in Figure 1.

Title: Supplementary Data 8. (Supplementary Data 8-DEG_up_DNMT1OE_down_enrichment.csv)
Description:
Gene ontology (GO) – biological process analysis of all genes with increased expression in E14.5 *Sst-Cre/tdTomato/Dnmt1 loxP*^{2} samples compared to *Sst-Cre/tdTomato* cells as well as reduced expression in *Dnmt1* overexpressing neurons transdifferentiated from murine ESCs compared to their controls.

Title: Supplementary Data 9. (Supplementary Data 9-DEG bTel and Ctx and overlap)
Description:
Collection of genes that were differentially expressed between E14.5 *Sst-Cre/tdTomato/Dnmt1 loxP*^{2} and *Sst-Cre/tdTomato* cells prepared from the cerebral cortex. Moreover, the overlap of significantly upregulated genes in *Sst-Cre/tdTomato/Dnmt1 loxP*^{2} cells from the basal telencephalon and from the cortex are summarized. Moreover, gene ontology analysis (Biological process) details are depicted, performed with the set of genes that were commonly upregulated in FAC-sorted *Sst-Cre/tdTomato/Dnmt1 loxP*^{2} neurons from both compartments (basal telencephalon and cortex). Background is defined as all detected transcripts in both datasets (ShinyGO 0.81; http://bioinformatics.sdstate.edu/go/).

Title: Supplementary Data 10. (Supplementary Data 10-all Raw Data-final)
Description:
This supplementary Excel file contains raw data, detailed statistical information and links to the deposited raw data for each main and supplementary figure presented in this manuscript.

Supplementary Movie legends
Title: Supplementary Movies 1, 2
Description:
Migratory behavior of interneurons depicted in organotypic brain slices (350 µm) of E14.5 *Sst-Cre/tdTomato* (Suppl. Video 1) and *Sst-Cre/tdTomato/Dnmt1 loxP*^{2} Suppl. Video 2) embryos. Footages display an imaging period of 20 hours with 15 minutes per frame. TdTomato$^+$ cells are depicted in grey. Scale bar: 100 µm. MZ: marginal zone, CP: cortical plate, VZ: ventricular zone.

Title: Supplementary Movies 3-9
Description:
Seizure events during PTZ-induced convulsions in three-month-old *Sst-Cre/tdTomato*) and *Sst-Cre/tdTomato/Dnmt1 loxP*^{2} mice. All videos are depicted in real-time. Videos 6 and 7 display examples of small seizures of event I (whole body myoclonus until reaching a standing position with an upward stretched tail). In videos 8 and 7 mice are displaying small seizure events of type II (whole-body myoclonus with stretched body and stretched tail pointing towards the head). Event III of seizure types is depicted in video 10, characterized by several convulsions leading to short rearing episodes, falling over, and rolling with subsequent recovery of the individual. Videos 11 and 12 show examples of final tonic-clonic seizures (event IV) with whole-body convulsions culminating in heavy rearing and overstretched limbs.

Title: Supplementary Movie 10
Description:
Stereotypic behavior of an adult *Sst-Cre* strain mouse depicting repetitive movements characterized by mounting on the play tunnel, flipping over, and instant repetition of the procedure. Stereotypic

behavior can indicate general vulnerability to stress or autism-like symptoms. Footages are depicted in real-time.
