# Supplementary material for: DNMT1-mediated regulation of somatostatin-positive interneuron migration impacts cortical architecture and function
Source: Nat Commun. 2025 Jul 24;16:6834. doi: 10.1038/s41467-025-62114-0 (PMC12290092; doi:10.1038/s41467-025-62114-0)
Supplement: Supplementary file 23 — Reporting Summary [file 41467_2025_62114_MOESM23_ESM.pdf]

nature portfolio

Corresponding author(s): Geraldine Zimmer-Bensch; NCOMMS-24-59357-T

Last updated by author(s): Jun 23, 2025

Reporting Summary

Nature Portfolio wishes to improve the reproducibility of the work that we publish. This form provides structure for consistency and transparency in reporting. For further information on Nature Portfolio policies, see our Editorial Policies and the Editorial Policy Checklist.

Statistics

For all statistical analyses, confirm that the following items are present in the figure legend, table legend, main text, or Methods section.

n/a	Confirmed	
☐	☒	The exact sample size (*n*) for each experimental group/condition, given as a discrete number and unit of measurement
☐	☒	A statement on whether measurements were taken from distinct samples or whether the same sample was measured repeatedly
☐	☒	The statistical test(s) used AND whether they are one- or two-sided<br>*Only common tests should be described solely by name; describe more complex techniques in the Methods section.*
☐	☒	A description of all covariates tested
☐	☒	A description of any assumptions or corrections, such as tests of normality and adjustment for multiple comparisons
☐	☒	A full description of the statistical parameters including central tendency (e.g. means) or other basic estimates (e.g. regression coefficient) AND variation (e.g. standard deviation) or associated estimates of uncertainty (e.g. confidence intervals)
☐	☒	For null hypothesis testing, the test statistic (e.g. *F*, *t*, *r*) with confidence intervals, effect sizes, degrees of freedom and *P* value noted<br>*Give P values as exact values whenever suitable.*
☒	☐	For Bayesian analysis, information on the choice of priors and Markov chain Monte Carlo settings
☐	☒	For hierarchical and complex designs, identification of the appropriate level for tests and full reporting of outcomes
☒	☐	Estimates of effect sizes (e.g. Cohen's *d*, Pearson's *r*), indicating how they were calculated

Our web collection on statistics for biologists contains articles on many of the points above.

Software and code

Policy information about availability of computer code

Data collection

- coding and analysis strategy for CellChat Single Cells are provided following the link:
https://github.com/Vogel-lab/Reichard-2024_DNMT1-Mediated_Regulation-of_IN_Migration44

- coding and analysis strategy for Evidence Accumulation Task provided following the link:
https://github.com/NabbefeldG/MS_task_gerion_V2_5

-coding and analysis strategy for for the electrophysiological dataset are provided following the link
https://github.com/musall/dnmt1SOM

- Software:
  - ImageJ/Fiji
  - LAS X
  - Keyence BZ-X800
  - Particle Mesh Ewald method
  - BD FACSDiva Software, version 9.4
  - Langevin dynamics
  - Monte Carlo barostat
  - Matlab 2020b, MathWorks
  - Kilosort v2.5
  - Phyton v3.7

- SpikeInterface analysis toolbox
- bcl2fastq Illumina
- nf-core/rnaseq pipeline v3.12 implemented in Nextflow 23.10.0/24.10.5
- nf-core/methylseq pipeline v2.6.0/3.0.0 implemented in Nextflow 23.10.0/24.10.5
- Trim Galore 0.6.7
- STAR 2.7.9a
- Salmon v1.10.1
- DESeq2 v.1.32.0
- Nextflow 23.10.0/24.10.5
- FastQC (http://www.bioinformatics.babraham.ac.uk/projects/fastqc)
- Trimmomatic
- Bismark
- GENCODE VM23 dataset
- USCS LiftOver tool
- IGV
- Ensembl release 112
- AME algorithm provided within the MEME suite
- ShinyGO 0.80/0.82 with the biological processes' dataset
- nf-core/chipseq pipeline version 2.0.0 with the mm10 reference genome
- THOR with default settings
- MEME-ChIP
- CellRanger v7.0.0
- R v4.3.2/4.4.0/4.4.1running under Ubuntu 20.04.6 LTS
- Seurat v5.0.1.9001
- CellChat v2.1.25
- rtacklayer
- Trailmaker
- Kilosort v2.5
- ANYmaze

Data analysis

- coding and analysis strategy for CellChat Single Cells are provided following the link:
https://github.com/Vogel-lab/Reichard-2024_DNMT1-Mediated_Regulation-of_IN_Migration

- costum code for Evidence accumulation task can be found here: https://github.com/NabbefeldG/MS_task_gerion_V2_5

-coding and analysis strategy for for the electrophysiological dataset are provided following the link
https://github.com/musall/dnmt1SOM

-Software:
- GraphPad PRISM
- ImageJ/Fiji
- BD FACSDiva Software, version 9.4
- CPPTRAJ
- Matlab 2020b, MathWorks
- Kilosort v2.5
- Phyton v3.7
- SpikeInterface analysis toolbox
- bcl2fastq Illumina
- nf-core/rnaseq pipeline v3.12 implemented in Nextflow 23.10.0/24.10.5
- nf-core/methylseq pipeline v2.6.0/3.0.0 implemented in Nextflow 23.10.0/24.10.5
- Trim Galore 0.6.7
- STAR 2.7.9a
- Salmon v1.10.1
- DESeq2 v.1.32.0
- Nextflow 23.10.0/24.10.5
- FastQC (http://www.bioinformatics.babraham.ac.uk/projects/fastqc)
- Trimmomatic
- Bismark
- GENCODE VM23 dataset
- USCS LiftOver tool
- IGV
- Ensembl release 112
- AME algorithm provided within the MEME suite
- ShinyGO 0.80/0.82 with the biological processes' dataset
- nf-core/chipseq pipeline version 2.0.0 with the mm10 reference genome
- THOR with default settings
- MEME-ChIP
- CellRanger v7.0.0
- R v4.3.2/4.4.0/4.4.1 running under Ubuntu 20.04.6 LTS
- Seurat v5.0.1.9001
- CellChat v2.1.25
- rtracklayer
- Trailmaker
-ANYmaze

For manuscripts utilizing custom algorithms or software that are central to the research but not yet described in published literature, software must be made available to editors and reviewers. We strongly encourage code deposition in a community repository (e.g. GitHub). See the Nature Portfolio guidelines for submitting code & software for further information.

Data

Policy information about availability of data

All manuscripts must include a data availability statement. This statement should provide the following information, where applicable:
- Accession codes, unique identifiers, or web links for publicly available datasets
- A description of any restrictions on data availability
- For clinical datasets or third party data, please ensure that the statement adheres to our policy

Data Availability
The data supporting the results of this study are available as described in the results and the method part as hyperlinks or were directly uploaded to corresponding data platforms which are also stated, respectively. We confirm there is no (privacy) conflict of sharing our data openly. Since our study did not used human/patient material there is no need to anonymize corresponding data to comply with ethical and legal standards. In addition to the original data generated during this research, secondary data used in the analysis are also referenced and can be accessed from the provided raw data overview, the mentioned data servers or via hyperlinks stated in the study. For further information regarding data access or requests, please contact Geraldine Zimmer-Bensch.

The sequencing datasets generated and/or analyzed for this study have been deposited in the Gene Expression Omnibus repository with the following accession numbers: GSE276510 [ChIP-seq], GSE298336 [snRNA-seq], GSE298865 [Methyl-seq], GSE276516 [RNA-seq bT] and GSE300332 [RNA-seq ctx]. The MERESCOPE datasets have been deposited in the Gene Expression Omnibus repository with the accession number GSE298591 [MERFISH]. Single cell RNA datasets from C57BL6/J embryos used for CellChat-based binding predictions are deposited under: GSE300648, GSE300649 and GSE291845 for the E14.5 dataset, and GSE291845, GSE300653 and GSE300678 for the E16.5 dataset. As this dataset is currently under consideration for publication in a different context, access is temporarily restricted and can be granted upon request by contacting Prof. Tanja Vogel (tanja.vogel@anat.uni-freiburg.de). The MD simulation data has been deposited in the MDposit IRB node under the project number A022O [https://irb-dev.mddbr.eu/#/id/A022O.1/overview]. Neuropixels recording data are available in a figshare repository [https://doi.org/10.6084/m9.figshare.28282838].

Research involving human participants, their data, or biological material

Policy information about studies with human participants or human data. See also policy information about sex, gender (identity/presentation), and sexual orientation and race, ethnicity and racism.

Reporting on sex and gender	n.a.
Reporting on race, ethnicity, or other socially relevant groupings	n.a.
Population characteristics	n.a.
Recruitment	n.a.
Ethics oversight	n.a.

Note that full information on the approval of the study protocol must also be provided in the manuscript.

Field-specific reporting

Please select the one below that is the best fit for your research. If you are not sure, read the appropriate sections before making your selection.

☒ Life sciences ☐ Behavioural & social sciences ☐ Ecological, evolutionary & environmental sciences

For a reference copy of the document with all sections, see nature.com/documents/nr-reporting-summary-flat.pdf

Life sciences study design

All studies must disclose on these points even when the disclosure is negative.

Sample size	All samples sizes used and analyzed are described for each Figure (captions) and/or in the corresponding methods part. Detailed information on the sample size determination can be found in the author checklist.
Data exclusions	All authors declare that no data were excluded from the study.
Replication	If applicable all biological or technical replicates are described in the corresponding Figure legends and/or in the related methods section.
Randomization	For randomization we used embryos/adult mice from different litters and from different parents. We only aimed to avoid incest breeding for terminated matings. For behavioral experiments we avoided using exclusively litter mates to artifically decrease variances of data. Experiments were done on different and independent days of conduction.
Blinding	Respective blinded analyses are stated in the Figure legends/captions and methods section. Mice and embryos were ID-coded.

Reporting for specific materials, systems and methods

We require information from authors about some types of materials, experimental systems and methods used in many studies. Here, indicate whether each material, system or method listed is relevant to your study. If you are not sure if a list item applies to your research, read the appropriate section before selecting a response.

Materials & experimental systems

n/a	Involved in the study	
☐	☒	Antibodies
☐	☒	Eukaryotic cell lines
☒	☐	Palaeontology and archaeology
☐	☒	Animals and other organisms
☒	☐	Clinical data
☒	☐	Dual use research of concern
☒	☐	Plants

Methods

n/a	Involved in the study	
☐	☒	ChIP-seq
☐	☒	Flow cytometry
☒	☐	MRI-based neuroimaging

Antibodies

Antibodies used	Primary antibodies:<br>- mouse anti-Calretinin (1:500; Swant, Switzerland, #6B3)

- rabbit anti-NPY (1:2500; Immunostar, U.S.A., #2940)
- rabbit anti-TBR1 (1:200; Abcam, U.S.A., #ab31940)
- rabbit anti-TBR2 (1:500; Abcam, U.S.A., #ab23345)
- rat anti-Somatostatin (1:100; Millipore, U.S.A., #MAB354)
- mouse anti-Parvalbumin (1:2000; Swant, Switzerland, #235)
- rabbit anti-DNMT1 (1:100; Santa Cruz, U.S.A., # sc20701)
- rabbit anti-CUX1 (CDP; 1:100, Santa Cruz, U.S.A., #sc13024)
- mouse anti-Nestin (1:100; Merck U.S.A., #MAB353)
- rabbit anti-ß-Tubulin III (1:500; Sigma Aldrich, U.S.A., #T2200)
- rabbit anti-ErB4 (1:1000; Proteintech, U.S.A., #22387-1-AP)

Secondary antibodies (all applyied 1:1000)
- Alexa488-goat anti-Rat IgG (Invitrogen, U.S.A., #A11006)
- Cy5-Goat anti-Rabbit IgG (Life Technologies, U.S.A., #A10523)
- A488-Donkey anti-Mouse IgG (Jackson, U.S.A., #15454150)
- Cy5-Goat anti-Mouse IgG (Jackson, U.S.A., #115175146)
- A488-Goat anti-Rabbit IgG (Life Technologies, U.S.A., #A11008)

Validation

All primary antibodies were tested following the manufacturer's provided information and data sheets, former studies, already established protocols in the laboratory, or during establishment including different dilutions/concentrations. Negative controls involved the incubation with secondary antibodies without the primary antibody.

Eukaryotic cell lines

Policy information about cell lines and Sex and Gender in Research

Cell line source(s)

cell lines:
- immortalized murine cerebellar granule (CB) cells: first described in Fossale et al. (2004; https://link.springer.com/article/10.1186/1471-2202-5-57), cells obtained Dr. Mukhran Khundadze (University Hospital Jena, Germany)

- murine embryonic stell cells (ESCs): first described in Saxena et al. (2020; https://www.semanticscholar.org/paper/Analysis-of-transcript-levels-of-a-few-candidate-in-Saxena-Maroju/987a863d41f64c7eb126bb58f020785c0f49f0e9) obtained from Prof. K. Naga Mohan Hyderabad University, Hyderabad, India)

primary cells (no cell lines):
- primary single cells for Cell-Chat (single cell signaling): single cells derived from the basal telencephalon from C57BL6/J mice embryonic day 14.5 and E16.5

- primary single cells of Sst-Cre/tdTomato and Sst-Cre/tdTomato/Dnmt1 loxP2 for FACS + bulk RNA sequencing and DNA methylation sequencing: single cells from the basal telencephalons and the cortex from embryos of both mouse strains were isolated from animals of embryonic day 14.5

- primary single cells MGE and cortex for cultivation: C57BL6/J: single cells were isolated from the MGE and the cortex from embryos at embryonic day 14.5

Authentication

The authors declare that the used cell lines and used single cells were not authenticated, except for the presence of tdTomato-expression in Sst-Cre/tdTomato- and Sst-Cre/tdTomato/Dnmt1 loxP2 embryos.

Mycoplasma contamination

The authors declare that none of the immortalized/generated cell lines were tested for mycoplasm contamination.

Commonly misidentified lines
(See ICLAC register)

n.a.

Animals and other research organisms

Policy information about studies involving animals; ARRIVE guidelines recommended for reporting animal research, and Sex and Gender in Research

Laboratory animals

Overview on number of used animals (embryonic and adult; Sst-Cre/tdTomato vs. Sst-Cre/tdTomato/Dnmt1 loxP2):
- PTZ experiments 13-17-week-old males: 10 vs. 10
- nestbuilding: 12-24-week-old males: 16 vs. 16
- evidence accumulation task and MWM 8-12-week-old males: 7 vs. 6
- neuropixels recordings 6-month-old males: 2 vs. 2
- cortical layer dimensions adult (6-month-old males): 3 vs. 3
- cortical layer distribution of tdTomato cells adult (6-month-old males): 3 vs. 3
- representative immunohistological co-stainings SST/NPY and SST/Calretinin adult: 4-month-old Sst-Cre/tdTomato male
- PV/tdTomato overlap adult 6-month-old males: 3 vs. 3
- E14.5 RNA seq: 23 vs. 14 basal telencephalon, 7 vs. 8 cortex
- E14.5 methylation seq: 15 vs. 11 basal telencephalon, 7 vs. 8 cortex
- E14.5 single-cell RNA seq-based CellChat binding predictions C57 embryos: 5

- E16.5 single-cell RNA seq-based CellChat binding predictions C57 embryos: 6
- E16.5 single-nucleus RNA seq incl. CellChat binding predictions: 2 vs. 2
- E16.5 MERFISH: 1 vs. 1
- E14.5 cortical dimensions, tdTomato+ cell distribution in entire hemispheres and in cortex, EOMES+ cell numbers, TBR+ cell numbers, DNMT1 KO validation: 3 vs. 3
- E16.5 cortical dimensions, tdTomato+ cell distribution in entire hemispheres and in cortex, EOMES+ cell numbers, TBR+ cell numbers: 4 vs. 4
- E18.5 cortical dimensions, layer thicknesses, areas CP and IZ-VZ, tdTomato+ cell distribution in cortex, EOMES+ cell numbers, TBR+ cell numbers: 4 vs. 4
- E14.5 SOX2+ cell numbers: 5 vs. 5
- E14.5 LCI organotypic brain slices tdTomato+ fluroescence signal increase in CP, radial migration, migrated pathlength, morphology analysis tdTomato+ cells: 3 vs. 3
- 14.5 LCI organotypic brain slices velocity of all cortical tdTomato+ cells and velocity tdTomato+ cells in area CP-VZ: 5 vs. 3
- 14.5 LCI organotypic brain slices average directional changes of tdTomato+ cells: 4 vs. 3

Mouse strains:
Transgenic mouse strains with a genetic C57BL/6J background (initially obtained from the University Hospital UKA Aachen, Germany) were used. Ssttm2.l(cre)Zjh/J x B6.CgGt(ROSA)26Sortml 4(CAG-tdTomato)Hze (Sst+/--Cre/tdTomato) served as control animals whereas Ssttm2.l(cre)Zjh/J x B6.CgGt(ROSA)26Sortm14(CAG-tdTomato)Hze x B6; 129S-Dnmt1tm2Jae/J (Sst-Cre+/-/tdTomato/Dnmt1 loxP2) were used as Dnmt1 knockout (KO) model. By crossing an Ssttm2.l(cre)Zjh/J strain (RRID:IMSR_JAX:013044, Jackson Laboratory, Bar Harbour, USA) with a tdTomato reporter mouse model (B6.CgGt(ROSA)26Sortml4(CAG-tdTomato)Hze, the latter obtained from Prof. Christian Hübner (University Hospital Jena, Germany)). An internal ribosomal entry site (IRES), a Cre-recombinase sequence, a polyA sequence, and a frt-flanked neo cassette were inserted into the 3′ untranslated region (UTR) of the Somatostatin (Sst) locus on chromosome 16, exclusively limiting the respective Cre-expression to SST-positive neurons. Mice of the tdTomato reporter strain had a tdTomato sequence combined with a loxP-flanked stop cassette in the Rosa26 locus. The Cre-recombinase transcription dependent on Sst-promoter activity resulted in an excision of the loxP-flanked stop cassette during recombination and a subsequent expression of tdTomato in Cre-positive Sst-expressing neurons. According to Jackson Laboratory we generated heterozygous mice that received the Cre-allele from the maternal side to avoid a potential instability of the Cre-recombinase activity during paternal germline recombination (jax.org/Strain/013044).

Wild animals

All authors state that no wild animals were used the current study.

Reporting on sex

- embryos isolated from C57BL6/J, Sst-Cre/tdTomato, and Sst-Cre/tdTomato/Dnmt1 loxP2 were used without consideration of their sex (identification would only have been possible post mortem through genotyping of presence of XX- or XY-chromosomes)

- for analyses considering adult Sst-Cre/tdTomato and Sst-Cre/tdTomato/Dnmt1 loxP2 mice: only males (between 3 and 6 month-old) were used to avoid high heterogeneity due to fluctuating estrogen levels in female mice

Field-collected samples

n.a.

Ethics oversight

All general procedures (housing, breeding) were carried out at the Institute of Zoology (Biology II, RWTH Aachen) and at the Center for Experimental Models and Transgenic Service (CEMT), University Medical Center Freiburg, in accordance with European Directive 2010/63/EU (ETS123), FELASA standards, and the German GV-SOLAS guidelines, with approval for animal husbandry according to §11 Animal Protection Act (TierSchG). For the procedures conducted at the RWTH Aachen, organ removal was performed under license 40168.A4, approved by the Institute for Laboratory Animal Research, University Hospital Aachen. Embryo isolation followed protocol 81-02.04.2019.A311, authorized by the LANUV (North Rhine-Westphalia). Behavioral experiments, including PTZ, Neuropixels recordings, Morris Water Maze, and (multi)sensory discrimination tasks, were conducted under protocol 81-02.04.2020.A175 and complied with the same European and national regulations. Procedures conducted on C57BL6/J mice at the University Medical Center Freiburg were approved by the Regierungspräsidium Freiburg under the licenses G19/125 and G21/082 and in accordance with §4 TierSchG.

Note that full information on the approval of the study protocol must also be provided in the manuscript.

Plants

Seed stocks

n.a.

Novel plant genotypes

n.a.

Authentication

n.a.

ChIP-seq

Data deposition

☒ Confirm that both raw and final processed data have been deposited in a public database such as GEO.

☒ Confirm that you have deposited or provided access to graph files (e.g. BED files) for the called peaks.

Data access links<br>*May remain private before publication.*	GSE276510
Files in database submission	DNMT1_Ctrl_FC_2_S3_R1_001.fastq.gz<br>DNMT1_Ctrl_FC_4_S7_R1_001.fastq.gz<br>DNMT1_Ctrl_FC_input_S9_R1_001.fastq.gz<br>DNMT1_Ctrl_input.bigWig<br>DNMT1_Ctrl_REP2.bigWig<br>DNMT1_Ctrl_REP4.bigWig<br>DNMT1_Efna5_FC_1_S2_R1_001.fastq.gz<br>DNMT1_Efna5_FC_3_S6_R1_001.fastq.gz<br>DNMT1_Efna5_FC_input__S10_R1_001.fastq.gz<br>DNMT1_Efna5_input.bigWig<br>DNMT1_Efna5_REP1.bigWig<br>DNMT1_Efna5_REP3.bigWig
Genome browser session<br>(e.g. UCSC)	*Provide a link to an anonymized genome browser session for "Initial submission" and "Revised version" documents only, to enable peer review. Write "no longer applicable" for "Final submission" documents.*

Methodology

Replicates	N = 2 biological replicates per group
Sequencing depth	single-end, 478.7 million reads across samples
Antibodies	ChIP-seq:<br>- anti-DNMT1 (BioAcademia, Japan, #70-201)<br>- normal rabbit IgG (Merck Millipore, U.S.A., #12-370)
Peak calling parameters	THOR, default settings: Allhoff, M., Seré, K., F Pires, J., Zenke, M. & G Costa, I. Differential peak calling of ChIP-seq signals with replicates with THOR. Nucleic Acids Res 44, e153 (2016).
Data quality	Only differential peaks called by THOR on default settings were used in this study, with a cutoff of -log10(pval) at 1.4
Software	Described in results and methods.

Flow Cytometry

Plots

Confirm that:

☒ The axis labels state the marker and fluorochrome used (e.g. CD4-FITC).

☒ The axis scales are clearly visible. Include numbers along axes only for bottom left plot of group (a 'group' is an analysis of identical markers).

☒ All plots are contour plots with outliers or pseudocolor plots.

☒ A numerical value for number of cells or percentage (with statistics) is provided.

Methodology

Sample preparation	For FACS-mediated enrichment of E14.5 Sst-Cre/tdTomato cells, the whole basal telencephalon was prepared from the embryos and subjected to cell dissociation. Nuclease-free reaction tubes were used during isolation and long-term storage of the resulting material. The basal telencephaloni were collected in cold HBSS (w/ phenol red, w/o calcium, w/o magnesium)/0.65% D-glucose; 4 µg/µL (600 U) of DNAse I (AppliChem GmbH, Germany). After treatment with 0.04% trypsin for 17 min at 37 °C, HBSS was replaced by DMEM with additional L-glutamine and 4.5 g/L D-glucose, 10% FBS and 1% P/S to stop the trypsinization. Subsequently, the cells were pelletized, resuspended and triturated in cold HBSS (w/o phenol red, w/o calcium, w/o magnesium)/0.65% D-glucose, before being filtered through a nylon gauze (pore size 140 µm, Merck, U.S.A.) for FACS.<br><br>FACS was performed by the Flow Cytometry Facility (FCF, University Hospital RWTH Aachen, Germany). Respective parameters for the procedure using a BD FACS Aria Fusion (BD Biosciences, U.S.A) were defined as follows: 5-laser (FCS, SSC, PE, BV421), 18-color (3-6-2-4-3). TdTomato-positive cells were either collected in 100 µL of cold TRIzol™ (Thermo Fisher

Scientific, U.S.A.) for subsequent RNA sequencing or in 100 µL of cold HBSS (w/o phenol red, w/o calcium, w/o magnesium)/0.65% D-glucose for further processing for DNA methylation analysis. Finally, all samples were stored at –80°C.

Instrument	BD FACS Aria Fusion (Config: 5-laser, 18-color (3-6-2-4-3))
Software	BD FACSDiva Software, version 9.4
Cell population abundance	Since we aimed to collect all td-Tomato positive cells which was a small fraction, we did a purity check with Cre-negative samples. Values were always >99%.
Gating strategy	(FSC vs SSC), Single cells, Live cells (Dapi negative), Tomato positive cells (gates were set in accordance to a Cre/tdTomato-negative sample)

☒ Tick this box to confirm that a figure exemplifying the gating strategy is provided in the Supplementary Information.
